# Petrogenesis of volcanic rocks from the Quaternary Eifel volcanic fields, Germany: detailed insights from combined trace-element and Sr–Nd–Hf–Pb–Os isotope data

**DOI:** 10.1007/s00410-024-02137-w

**Published:** 2024-05-09

**Authors:** Mike W. Jansen, Carsten Münker, Josua J. Pakulla, Eric Hasenstab-Dübeler, Christian S. Marien, Toni Schulz, Maria Kirchenbaur, Kathrin P. Schneider, Robin Tordy, Vera Schmitt, Frank Wombacher

**Affiliations:** 1 Institut für Geologie und Mineralogie, Universität zu Köln, Zülpicher Str. 49B, 50674 Cologne, Germany; 2 Department für Lithosphärenforschung, Universität Wien, Josef-Holaubek 2 (UZA II), 1090 Vienna, Austria; 3 Institut für Geologie, Mineralogie, und Geophysik, Ruhr-Universität Bochum, Universitätsstraße 150, 44801 Bochum, Germany

**Keywords:** Quaternary volcanism, Carbonated eclogite, Lithosphere, Plume, Central European Volcanic Province, Eifel

## Abstract

Quaternary rocks from the East and West Eifel volcanic fields in western Germany are a key suite of intraplate volcanic rocks that can provide insights into volcanism of the Central European Volcanic Province (CEVP) and into continental intraplate volcanism in general. We present a comprehensive dataset for Eifel lavas including isotope as well as major and trace element data for 59 samples covering representative compositions of the different volcanic fields. In line with previous studies, the lavas are all SiO_2_-undersaturated, alkaline-rich and mainly comprise primitive basanites, melilitites, and nephelinites (Mg# ≥ 57). Geochemical compositions of samples from both volcanic subfields display distinct differences in their trace-element as well as radiogenic isotope compositions, largely confirming previous subdivisions. Coupled trace-element and radiogenic Sr–Nd–Hf–Pb–Os isotope compositions can now provide firm evidence for spatially heterogeneous mantle sources and compositionally distinct magmatic pulses. Within the West Eifel Field, Sr–Nd–Pb isotope compositions of the younger (≤80 ka), ONB-suite (olivine-nephelinite-basanite) are similar to FOZO (FOcal ZOne) or the EAR (European Asthenospheric Reservoir) and resemble compositions that have been previously reported from plume-sourced ocean island basalts (OIB). In marked difference, older (700 Ma to 80 ka) volcanic rocks from the F-suite (Foidite) in the West Eifel field and from the entire east Eifel Field tap a more enriched mantle component, as illustrated by more radiogenic Sr isotope (^86^Sr/^87^Sr up to 0.705408) and variable Pb isotope compositions (^206^Pb/^204^Pb = 18.61–19.70, ^207^Pb/^204^Pb = 15.62–15.67 and ^208^Pb/^204^Pb = 38.89–39.76). Combined trace-element compositions of ONB-suite samples are in good agreement with results from batch melting models suggesting a hybrid composition of Eifel magmas formed through mixing 10% of a FOZO-like melt with 90% of a DMM-like melt, similar to melts from the Tertiary HEVF. However, radiogenic Sr–Nd–Pb isotope compositions of F-suite and EEVF and some ONB lavas require the admixture of melts from lithospheric mantle sources. Elevated Nb/Ta and Lu/Hf ratios in combination with variable ^187^Os/^188^Os ratios can now demonstrate the presence of residual carbonated eclogite components, either in the lithosphere or in the asthenospheric mantle. Finally, by combining geochemical and temporal constraints of Tertiary and Quaternary volcanism it becomes evident that CEVP volcanism in central and western Germany has resulted from compositionally distinct magmatic pulses that tap separate mantle sources. Although the presence of a mantle plume can neither be fully confirmed nor excluded, plume-like melt pulses which partially tap carbonated eclogite domains that interact to variable extents with the lithosphere provide a viable explanation for the temporal and compositional cyclicity of CEVP volcanism.

**Supplementary Information:**

The online version contains supplementary material available at 10.1007/s00410-024-02137-w.

## Introduction

Volcanism within the Central European Volcanic Province (CEVP) commenced in the late Cretaceous (<65 Ma) and continued throughout the Tertiary until present day (Wilson and Downes 1991). The CEVP forms a 750 km long volcanic belt that extends across Europe from France to Poland and Hungary. Eruptive volcanism has been dominated by lavas that occur in spatially distinct, individual volcanic fields and comprise scoria cones, maars and associated lava flows as well as scattered necks and plugs (Wörner et al. 1986; Wilson and Downes 1991; Cohen and Waters 1996; Lustrino and Wilson 2007). Focusing on northern central Europe, several studies have investigated the geochronological, geochemical and petrological characteristics of pre-Pleistocene volcanism within the Siebengebirge (e.g., Todt and Lippolt 1980; Kolb et al. 2012; Schneider et al. 2016; Jung et al. 2011), Vogelsberg (Jung and Masberg 1998; Haase et al. 2004; Jung et al. 2011), Rhön (Jung and Hoernes 2000; Jung et al. 2005), Westerwald (Haase et al. 2004), Urach-Hegau (e.g., Hegner et al. 1995), Heldburg (Abratis et al. 2015; Pfänder et al. 2018) and the Hocheifel (Jung et al. 2006; Fekiacova et al. 2007). One of the major questions driving ongoing research on CEVP volcanism is, as to whether the mantle sources of the primary magmas are dominated by the convecting asthenosphere or the overlying lithospheric mantle. Based on geochemical compositions that are broadly similar to Ocean Island Basalts (OIB) (Hofmann 2003; Stracke et al. 2005; Mertz et al. 2015) and on geophysical evidence (e.g., Granet et al. 1995; Hoernle et al. 1995; Goes et al. 1999), it has been argued that CEVP volcanism might be linked to mantle plume activity. However, although several seismological studies reported anomalous mantle temperatures and low-velocity anomalies in the mantle underneath the CEVP (e.g., Ritter et al. 2001; Ritter and Christensen 2007), it yet remains ambiguous, if one or multiple mantle plumes exist underneath central Europe and from which mantle depths upwelling material is sourced (e.g., Granet et al. 1995; Goes et al. 1999, 2000; Ritter et al. 2001; Ritter and Christensen 2007). Notably, the potential mantle temperatures are also below classical plume-related localities like Hawaii (Putirka 2005). As an alternative to plume models, OIB-like compositions found in continental intraplate volcanic rocks have been explained by the entrainment of mantle lithosphere and crustal materials en route to the surface, given that continental intraplate basalts erupt through a variably thick lithosphere (e.g., Wilson and Downes 1991). As most of the volcanic fields within the CEVP are pertaining to a NNE-trending rift system that includes the Rhone, Limagne, Bresse, Rhine, Ruhr, and Leine Graben structures (e.g., Wilson and Downes 1991) volcanism has alternatively been suggested to originate from passive upwelling and extension-induced adiabatic decompression melting (e.g., Bogaard and Wörner 2003; Fekiacova et al. 2007; Kolb et al. 2012).

Notably, state of the art geochemical data for volcanic rocks from the Quaternary Eifel volcanic field in western Germany, one of the key volcanic suites to better understand magmatism of the CEVP, were missing so far. In this study, we aim to fill this gap by presenting a comprehensive trace element and Sr–Nd–Pb–Hf–Os isotope dataset for representative volcanic rocks from the Quaternary Eifel volcanic field that helps to evaluate the different petrogenetic models being proposed.

## Regional geological setting and sample selection

The Eifel volcanic field (EVF) is located within the Rhenish Massif of the Variscian orogen, an uplifted lithological block that is cut by the Upper- and Lower Rhine Graben in Western Germany. The basement of the EVF is built up by upper-crustal Devonian and Triassic sediments, a mid-crustal metamorphic basement (e.g., mica-schists) and lower crustal mafic rocks in granulite facies (Stosch and Lugmair 1984, 1986; Wörner et al. 1985, 1986). Major structural domains of central Europe (e.g., Rhenish Massif) that build the basement of the EVF have been emplaced during the Variscian orogeny (360 to ~300 Ma) in the Upper Devonian and Lower Carboniferous. The formation of these structural domains results from the accretion of several microcontinents during the collision of Laurasia and Gondwana (Wilson and Downes 1991).

The magmatic activity within the CEVP and also in the EVF have been previously ascribed to mirror the extensive tectonic history which caused lithospheric flexure and passive upwelling that led to adiabatic decompression and partial melting of both the upper asthenospheric and lithospheric mantle (Wilson and Downes 1991). However, identification of seismologically anomalous structures beneath the CEVP that were interpreted as single or multiple mantle-plumes have also been used to argue for lower mantle components in the sources of CEVP and therefore EVF melts (e.g. Ritter et al. 2001; Ritter and Christensen 2007). Collectively, it has been proposed that the primary magmas of the CEVP result from a complex mixture involving overall heterogeneous asthenospheric- and lithospheric mantle sources (e.g., Wörner et al. 1986; Wilson and Downes 1991; Jung et al. 2006; Kolb et al. 2012; Pfänder et al. 2012, 2018; Schneider et al. 2016).

Based on spatial, temporal and compositional patterns, the EVF can be further subdivided into a Tertiary Hocheifel volcanic field (HEVF, ~44–35 Ma) (Fekiacova et al. 2007) and two Quaternary subfields, the West Eifel volcanic field (WEVF, ~700–10.8 ka) and the East Eifel volcanic field (EEVF, 500–12.9 ka) (Schmincke 2007 and references therein). The older WEVF which is dominated by scoria cones and maars, has an aerial extent of ~600 km^2^ and extends form Ormont in the northwest to Bad Bertrich in the southeast. In contrast, the EEVF (areal extent of ~400 km^2^) comprises four phonolitic centers that can be distinguished in space and time: Kempenich (500–450 ka; according to Schmincke 2007), Rieden (450–300 ka; Viereck 1984; Nowell et al. 2006 and references therein), Wehr (215–151 ka; van den Bogaard et al. 1989) and Laacher See (13 ka; Reinig et al. 2021).

The EEFV contains a few maars but numerous scoria cones and voluminous lava flows (e.g., Schmincke 2007 and references therein) that are often slightly differentiated in composition. Primitive volcanic rocks of the WEVF and EEVF are all SiO_2_-undersaturated, mafic, and alkaline and the WEVF rocks can be subdivided into distinct petrogenetic suites, (1) a suite mainly composed of nephelinites and melilitites (F-suite) and an olivine-nephelinite-basanite-suite (ONB-suite) (Mertes and Schmincke 1985). The eruptive products of the WEVF differ from those of the EEVF by displaying more mafic and silica-undersaturated magmatic compositions and a greater abundance of mantle xenoliths (e.g., Schmincke 2007 and references therein). The occurrence and distribution of both petrogenetic suites in the WEVF appears to be time-dependent (Mertes and Schmincke 1985; Mertz et al. 2015). Magmas of F-suite affinity are older than 480 ka, whereas ONB-suite magmas are restricted to the younger magmatic episode between 81 and 11 ka (Mertz et al. 2015). Within the EEVF, mafic magmas generally resemble the compositional spectrum reported for the F-suite in the WEVF.

Considering isotope studies, previously analyzed radiogenic Sr–Nd–Hf–Pb isotope compositions of EVF lavas have indicated a contribution of mantle sources with characteristics similar to those of enriched Mid-Ocean-Ridge Basalt (E-MORB), HIMU/FOZO, EM I and EM II-type OIB to both the Quaternary and Tertiary volcanic fields (e.g., Wörner et al. 1986; Jung et al. 2006; Fekiacova et al. 2007) as well as to the CEVP in general (e.g., Jung and Masberg 1998; Jung and Hoernes 2000; Jung et al. 2006, 2011; Fekiacova et al. 2007; Lustrino and Wilson 2007; Lustrino 2011; Pfänder et al. 2012, 2018). Based on the overall compositional similarity of volcanic rocks erupted within the CEVP, several studies have further suggested a common, asthenospheric mantle reservoir underneath central Europe that has been named European Asthenospheric Reservoir (EAR) (Cebria and Wilson 1995), Low Velocity Component (LVC) (Hoernle et al. 1995), Common Mantle Reservoir (CMR) (Lustrino and Wilson 2007), or Prevalent Mantle (PREMA, Wörner et al. 1986), which is thought to contribute the HIMU-FOZO flavour. Although the proposed compositions for this component vary in detail, it is believed to be geochemically rather depleted and to represent a common mantle endmember located within the asthenosphere (e.g., Lustrino and Wilson 2007). Given that the inferred composition of this reservoir is broadly similar to the defined composition of FOZO (Hart et al. 1992), we will refer to this acronym throughout this study. Wörner et al. (1986) have suggested that crustal assimilation has only played a minor role within EVF volcanism, and that the variability of isotope compositions rather mirrors a heterogeneous composition of mantle underneath the EEVF. Compared to other volcanic provinces within central Europe, only limited trace-element and radiogenic isotope data are available for Quaternary EVF volcanism. In addition, most previous studies focused on Tertiary volcanic rocks (e.g., Jung et al. 2006; Fekiacova et al. 2007) and only included limited data for Quaternary volcanism (Wörner et al. 1986). Thus, the relationships between Tertiary and Quaternary volcanism and the difference in mantle sources involved remain poorly constrained.

Extensive studies on mantle xenoliths have shown that compositions of clinopyroxenite and hornblendite veins share nearly the same diversity in trace-element and radiogenic isotope compositions as all of the primary mantle derived Quaternary Eifel lavas (e.g., Witt-Eickschen and Kramm 1998; Witt-Eickschen et al. 2003). As the mantle xenoliths lack a pristine DMM signature, it has been inferred that a metasomatic overprint of the lithospheric mantle underneath the Eifel has occurred in the Tertiary and possibly during the Variscian orogeny (Witt-Eickschen and Kramm 1998; Witt-Eickschen et al. 2003). Witt-Eickschen et al. (2003) have suggested that the compositional diversity of xenoliths in Eifel lavas has resulted from the interaction of primary (DMM-like) asthenospheric melts and a highly enriched lithospheric mantle. This is broadly similar to the later model of Mertz et al. (2015) who also envisioned mixtures of asthenospheric and lithospheric melts to account for the varying geochemical compositions of west Eifel ONB- and F-suites. Moreover, based on detailed age constraints, a spatial boundary separating >480 ka F-suite and <80 ka ONB-suite volcanism was mapped out and is apparently also supported by seismological data (Mertz et al. 2015). Mertz et al. (2015) argued that the ONB-suite overlaps with a surface-projected low-velocity anomaly, indicative of a plume-like source within the asthenosphere. Further to this, the areal extent of F-suite volcanism was seen as being linked to an intensive thermal erosion of the lithosphere. The geochemical compositions of ONB-suite volcanic rocks were therefore proposed to tap the asthenospheric source reservoir, whereas F-suite rocks are compositionally more similar to the metasomatized lithospheric mantle (Mertz et al. 2015). This view will be critically evaluated below.

**Fig. 1:**
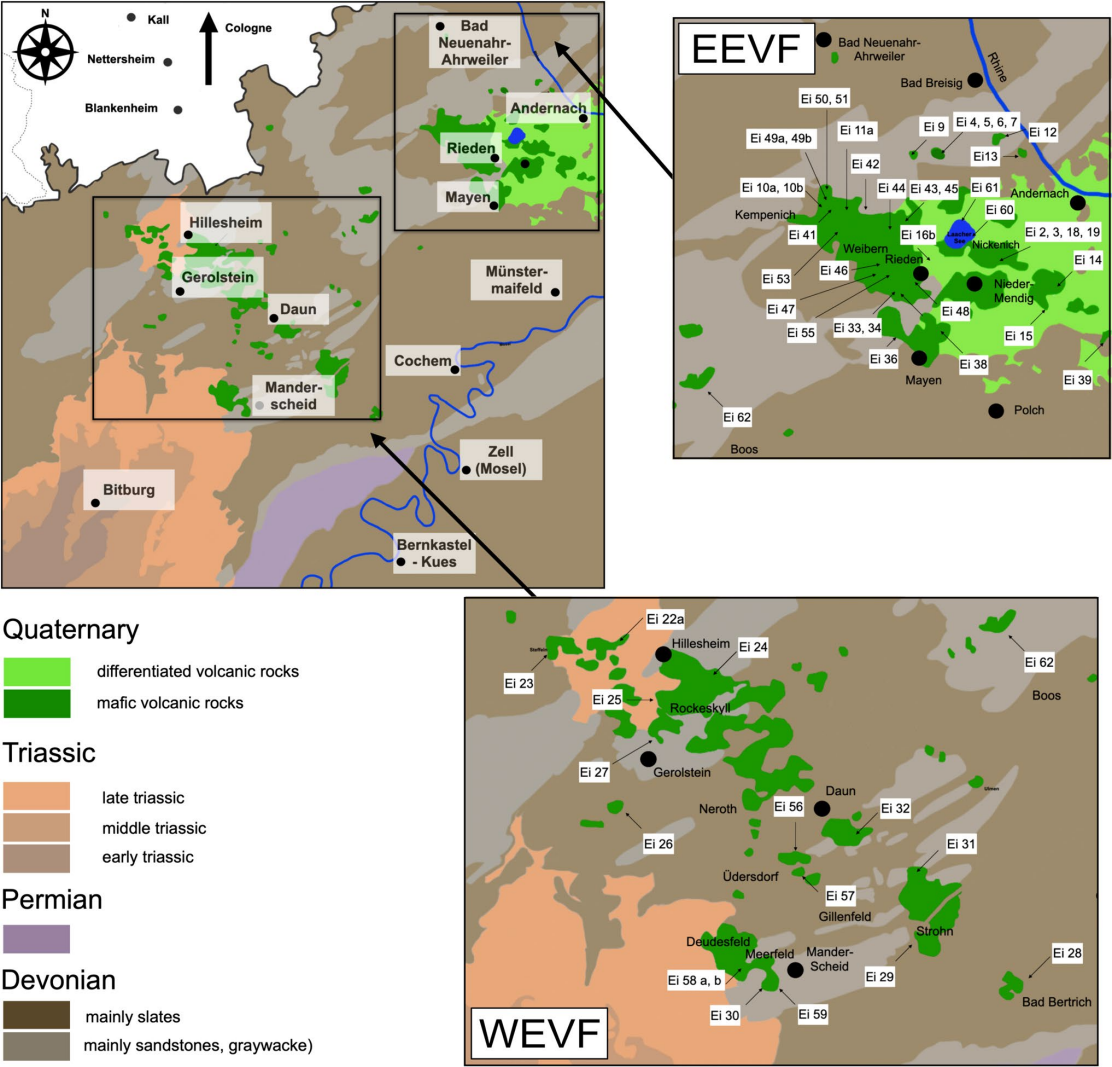
Simplified geological map showing the regional extent of the Quaternary Eifel volcanic fields (EVF) and the sample sites investigated

For this study we have selected 59 samples that cover both Quaternary volcanic subfields, the WEVF, and the EEVF, respectively. Moreover, we have re-sampled several volcanic edifices (e.g., scoria cones, lava flows, dikes) that have been previously characterized (Mertes 1983; Mertes and Schmincke 1985) and were used to define the F-, FE- and ONB-suite petrogenetic groups (Mertes and Schmincke 1985). We have also sampled highly differentiated phonolitic domes from the EEVF, focusing on the Rieden volcanic complex. The sampling sites are shown in a simplified geological map in Fig. 1, details on the sampling sites, including coordinates can be found in Table S1. In the field, sampling has been focused on fresh, massive blocks of least vesicular lava. Following further alteration control in thin sections, we analyzed all samples for their major- and trace element compositions as well as their ^87^Sr/^86^Sr, ^143^Nd/^144^Nd, ^176^Hf/^177^Hf, ^206,207,208^Pb/^204^Pb isotope compositions. Selected samples were also analyzed for their ^187^Os/^188^Os isotope compositions.

## Methods

Major elements were analyzed by XRF on fused disks using a Phillips PW 2400 spectrometer at the University of Cologne or with a Rigaku ZSX Primus IV at the Ruhr University Bochum (Table S1). Trace elements were measured with a quadrupole ICP-MS using an Agilent 7500 cs mass spectrometer at the University of Kiel following the analytical protocol by Garbe-Schönberg (1993) or with a Thermo Fischer iCap-Q at the University of Cologne following the analytical protocol of Pakulla et al. (2023). For trace element analysis, a representative sample split of ~100 mg was dissolved in a 1:1 14N HNO_3_/24N HF mixture (6 ml) in closed Savillex^®^ PFA Beakers on a hot plate at 130–150 °C for at least 2 days. To ensure the complete digestion of more differentiated samples, the samples were dried down and re-digested in Parr^®^ bombs for 24 h at 180 °C using the same acid mixture as for the tabletop digestion (see Hoffmann et al. 2010). Following several drydown steps in 14N HNO_3_ the samples were dissolved in 10 ml 0.28 N HNO_3_ for final measurements.

For Sr–Nd–Hf isotope analysis ~100 mg of sample powder were leached in cold 6N HCl for 15 min. After repeated washing steps, the samples were digested as described above. Following the procedure of Münker et al. (2001) a clean Hf and a matrix cut were obtained using Eichrom Ln-Spec ion exchange resin. Further separation of Sr and Nd from the matrix cut was done using AG 50W-X8 and Eichrom Ln-Spec ion exchange resins (Pin and Zalduegui 1997).

For Pb isotope measurements, ~250 mg of 3–4 mm sized rock chips were hand-picked and subsequently leached in 3N and 6N HCl for 1 h, respectively (Schuth et al. 2011). Following a digestion step in a 3:1 cHF-cHNO_3_ mixture, Pb was purified following the methods outlined in Korkisch and Hazan (1965) using AG 1-X8 anion resin.

For HSE (highly siderophile elements) and ^187^Re–^187^Os analysis, ~0.5–2 g of sample powder was spiked with a mixed ^185^Re–^190^Os–^192^Ir–^194^Pt tracer, digested in 7 ml of a 5:1 cHNO_3_-cHCl mixture and treated at 270 °C between 100 and 130 bar in an Anton Paar high-pressure asher (HPA) for 12 h. Osmium was then extracted using the liquid extraction procedure described by Cohen and Waters (1996) and further purified using a H_2_SO_4_/H_2_CrO_4_ micro-destillation (Birck et al. 1997). Following the Os extraction, the aqua regia fraction which contains all other HSEs and undissolved components was dried down and re-dissolved in 24N HF at 120 °C. After drydown the HSEs were separated by using AG 1-X8 resin, following published protocols by Rehkämper and Halliday (1997) and Coggon et al. (2013).

Strontium-Nd-Hf-Pb isotope measurements were performed on a Thermo Fisher Neptune MC ICP-MS at the University of Cologne. The measured Sr–Nd isotope data were mass bias corrected using the exponential law relative to ^86^Sr/^88^Sr = 0.1194 and ^146^Nd/^144^Nd = 0.7219, respectively. The measured values are reported relative to the accepted values for NBS 987 and La Jolla (^87^Sr/^86^Sr = 0.710240 for NBS 987 and ^143^Nd/^144^Nd = 0.511859 for La Jolla). Hafnium isotope data are reported relative to the Münster AMES standard which has a ^176^Hf/^177^Hf of 0.282160, indistinguishable from the JMC 475 Hf standard (Blichert-Toft and Albarède 1997). The external long-term reproducibility is ± 1 ε-unit for Sr and ± 0.4 ε-units for Nd and Hf, respectively. Procedural blanks were typically <100 pg for Hf and <300 pg for Nd and Sr. For Pb isotope analysis, doped NBS 991 Tl was used for mass bias correction, assuming a ^205^Tl/^203^Tl ratio of 1.0083 for doped samples and standards (Hirata 1996; Albarede et al. 2004; Kirchenbaur et al. 2011). Procedural blanks of Pb isotope measurements were typically better than 40 pg.

Osmium isotope compositions were measured using a Thermo Finnigan Triton Thermal Ionization Mass Spectrometer at the Department of Lithospheric Research at the University of Vienna. Osmium was measured as OsO^3−^, applying a peak hopping sequence using the Triton SEM detector. Oxygen corrections have been performed using ^17^O/^16^O = 0.0003866 and ^18^O/^16^O = 0.00203406 (Van Acken et al. 2011). Isobaric interferences of ^187^Re on ^187^Os and mass fractionation were conducted offline, using ^192^Os/^188^Os = 3.083.

Rhenium, Os, Pt and Ir concentrations were measured by isotope dilution on a Thermo Element ICP-MS at the Steinmann Institute at the University of Bonn. Instrumental drift was monitored and corrected using a 1 ppb in-house multi-element HSE standard solution with ratios of ^185^Re/^187^Re = 0.5986, ^191^Ir/^193^Ir = 0.5957 and ^198^Pt/^195^Pt = 0.2117. Additional isobaric Hf-oxide interferences on Ir and Pt were monitored and corrected offline. Analytical blanks were in a range of 0.2–0.5 pg (Os), 3–6 pg (Re), 0.5–1 pg (Ir) and 10–30 pg for Pt. Blank corrections were applied in all cases, although in most cases these are insignificant. Analytical quality was monitored with repeated measurements of reference materials OKUM (komatiite; Meisel et al. 1996) processed alongside the samples. All analyses of reference materials reproduce certified values within 2σ error.

## Results

### Major- and trace-element compositions

**Fig. 2:**
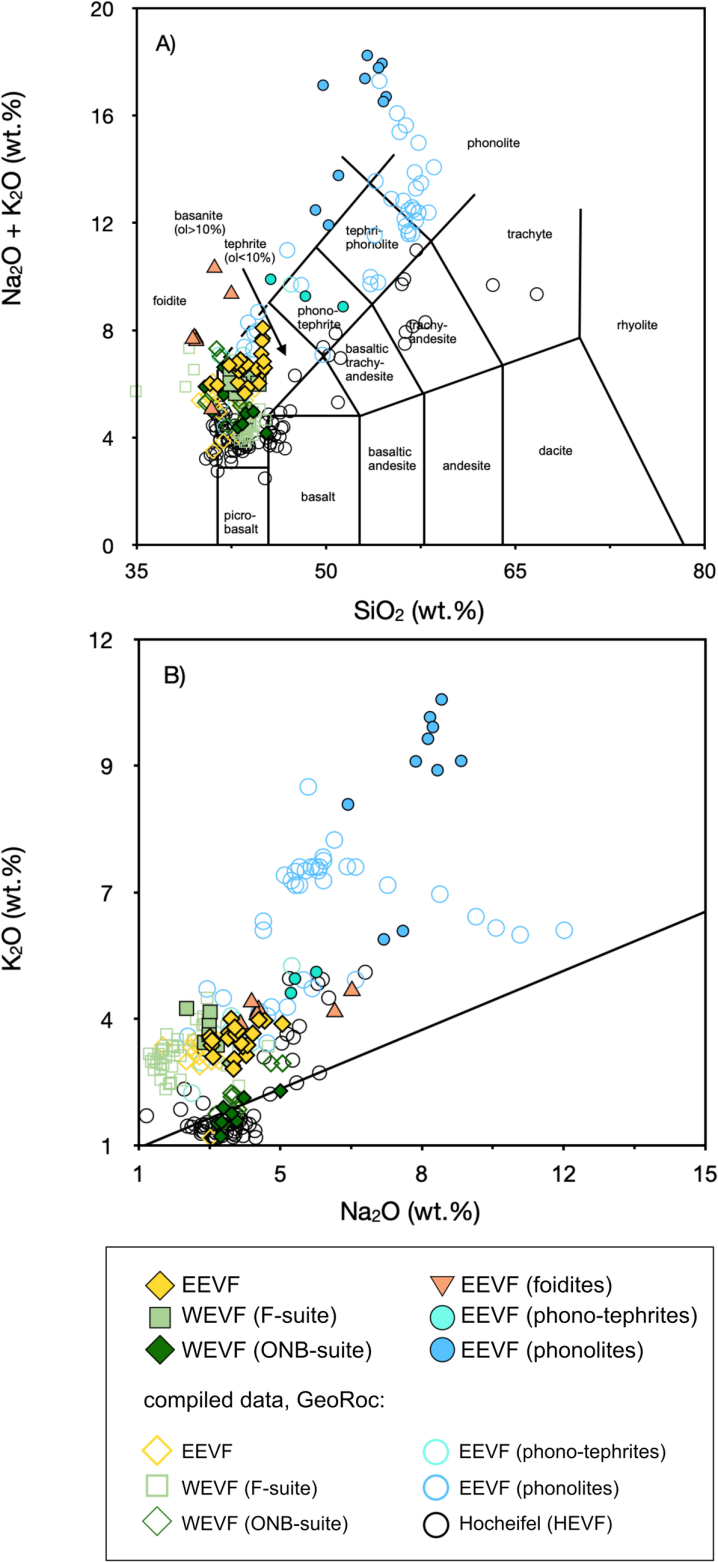
**A** Total Alkali (Na_2_O + K_2_O) versus Silica (SiO_2_) (TAS) diagram for the samples investigated within this study. The samples are characterized as silica undersaturated and alkaline (SiO_2_ = 39.4–54.7 wt.%; Na_2_O + K_2_O = 4.22–19.2) with most samples plotting in the basanite/tephrite fields. Foiditic and phonolitic volcanic rocks are only found within the EEVF. **B** Plot of Na_2_O (wt.%) versus K_2_O (wt.%), defining clear compositional groups. The samples of this study follow different magmatic trends. Samples from the EEVF are potassium enriched, while WEVF volcanic rocks display a bimodal distribution. Samples from the F-suite are potassic and compositionally overlap with the EEVF. In contrast, ONB-suite samples are more sodic. Compositional fields and literature data have been compiled using data from the GeoRoc database (https://georoc.eu/georoc/new-start.asp)

**Fig. 3:**
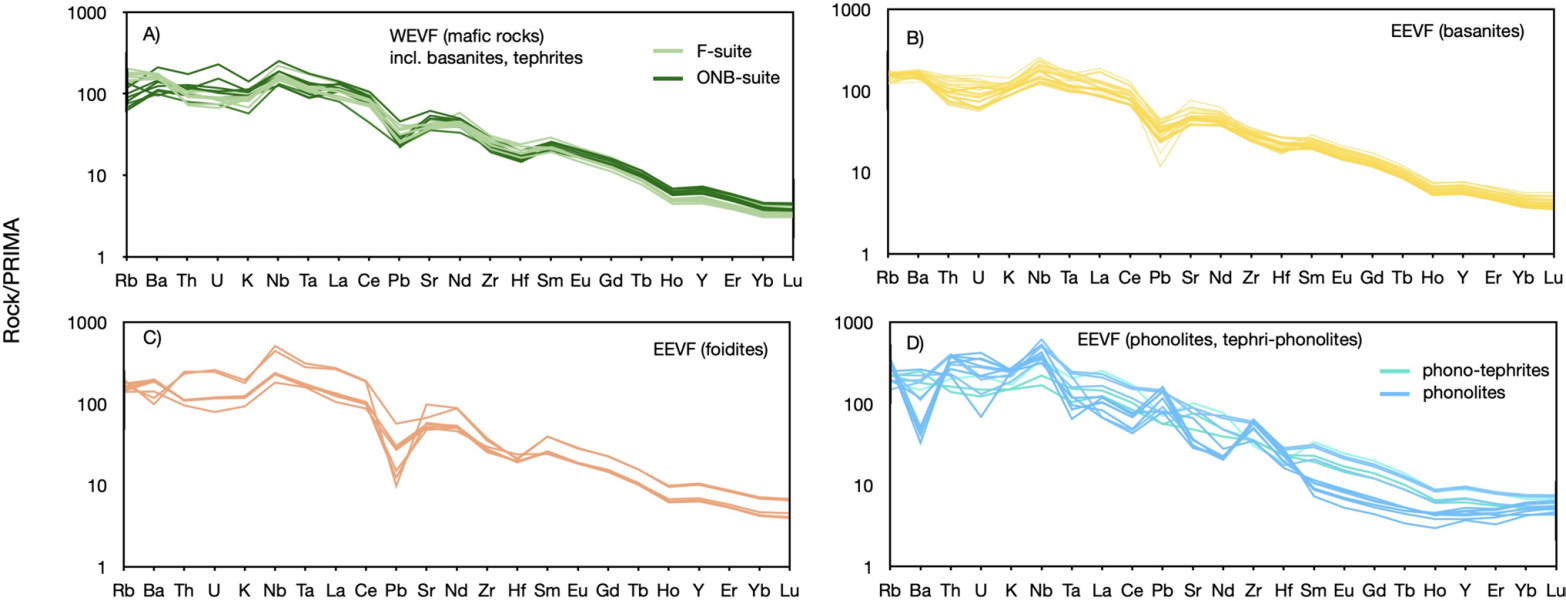
Primitive mantle-normalized trace element diagrams for samples from the Eifel volcanic fields. Illustrated in figures **A** and **B** are mafic samples of the WEVF and the EEVF that display strong depletions/enrichments of Ba, Nb and Pb and negative Zr–Hf anomalies. Detailed comparisons of both volcanic fields indicate that K and Pb anomalies are more pronounced in samples from the EEVF (**B**) compared to the WEVF (**A**), in foidites compared to basanites within the EEVF (**B**, **C**) and in the ONB-suite compared to the F-suite within the WEVF (**A**). In contrast, trace element patterns of differentiated rocks (phono-tephrites and phonolites, **D**) display patterns indicative of fractional crystallization with negative Ba and Ta and positive Nb, Pb and Zr anomalies. A negative Eu anomaly is not recognized and absent in all differentiated melts (**D**). Normalization values after Palme and O'Neill (2014)

All data of this study are listed in Tables S1–S5. Major and trace element compositions are listed in Table S1. The samples analyzed in this study were classified after Le Maitre (2002). First samples with over 20% of normative nepheline were classified as nephelinites, prior to further discriminated between foidites, basanites, tephrites, phono-tephrites, and phonolites using the TAS diagram (Fig. 2). Most phonolites plot outside the TAS phonolite field due to a marked enrichment in alkaline elements, but still fall into the phonolite field using the Streckeisen classification. We further distinguished the rocks plotting into the foidite field into more Mg-rich melilitites and silica-rich phonolitic foidites, following Le Maitre (2002). For simplicity we grouped the samples into 6 groups. In this regard the nephelinites and melilitites were combined to “EEVF foidites”, phono-tephrites and tephrites were combined to “EEVF phono-tephrites”, and phonolitic foidites and phonolites were combined to “EEVF phonolites”. Further, basanites from the EVF were divided into “EEVF”, “WEVF ONB-suite”, and “WEVF F-suite”. In general, samples from the EEVF are more K-rich compared to WEVF samples that themselves show a bimodal distribution. While samples from the F-suite are potassic and overlap with the compositions of the EEVF, the samples from the ONB-suite are more sodic. In major element variation diagrams (Fig. S1), samples of both fields reveal negative co-variations of SiO_2_, Al_2_O_3_, K_2_O, and Na_2_O- and positive co-variations of CaO, TiO_2_, and Fe_2_O_3total_ vs. MgO. Volcanic rocks of both fields can be distinguished based on MgO compositions, where WEFV samples are typically characterized by higher MgO concentrations (MgO = 10.1–14.5%; Mg# = 64–74), compared to EEVF samples (MgO = 6.57–10.6 Mg# = 58– 71). This is further underlined by positive co-variations of MgO with Ni and Cr (Fig. S1), where WEVF magmas mark near-primitive compositions with higher MgO and generally elevated Ni (88.0–385 ppm) and Cr concentrations (189–580 ppm) compared to EEVF lavas (Ni = 53.4–222 ppm; Cr = 60.1–317 ppm). Except for two samples (Ei 27, Sarresdorf lava flow and Ei 57, Emmelberg), ONB-suite samples tend towards even higher MgO concentrations (MgO = 10.9–14.5; Mg# = 70–74), in accord with previous observations (Mertes and Schmincke 1985; Mertz et al. 2015). In primitive mantle-normalized trace element diagrams (Fig. 3), mafic samples display positive anomalies of Ba and Nb, negative Pb and K anomalies and also small negative U-Th and Zr-Hf anomalies. Detailed comparisons of both Eifel volcanic fields indicate that the K and Pb anomalies are more pronounced in samples from the EEVF compared to the WEVF, in foidites compared to basanites within the EEVF, and in the ONB-suite compared to the F-suite within the WEVF (Fig. 3A–C). In contrast, trace element patterns of more differentiated melts (phono-tephrites and phonolites) display features indicative of fractional crystallization with clear negative Ba and Ta as well as positive Nb, Pb and Zr anomalies relative (Fig. 3D). However, negative Eu anomalies are absent within the differentiated melts analyzed within this study.

ONB-suite samples display stronger negative anomalies for K (K/K* = 0.287–0.454) compared to F-suite and EEVF samples. Furthermore, mafic samples display high Zr/Hf (39.6–62.2) and Nb/Ta (15.9–23.5) that exceed ratios of primitive OIB (Zr/Hf up to 45, Nb/Ta ~15–16; Pfänder et al. 2007), but overlap with ratios previously reported for CEVP intraplate basalts (Kolb et al. 2012; Pfänder et al. 2012, 2018). Similarly, Ce/Pb (17.3–68.6) and Nb/U (27.7–61.4) ratios of mafic samples generally agree with previously reported ratios of OIB and the CEVP (e.g., Jung et al. 2006; Fekiacova et al. 2007; Mayer et al. 2018), but exceed the canonical ratios of Ce/Pb ~25 and Nb/U ~47 that have been inferred for OIBs (Hofmann et al. 1986). Additionally, REE ratios such as primitive mantle normalized (Dy/Yb)_N_ (1.59–1.96) and (La/Yb)_N_ (20.1–37.6) only display minor variations within mafic samples and are in good agreement with previously reported values for CEVP volcanic rocks (e.g., Haase et al. 2004; Jung et al. 2006; Mayer et al. 2018).

### Radiogenic isotope compositions

**Fig. 4:**
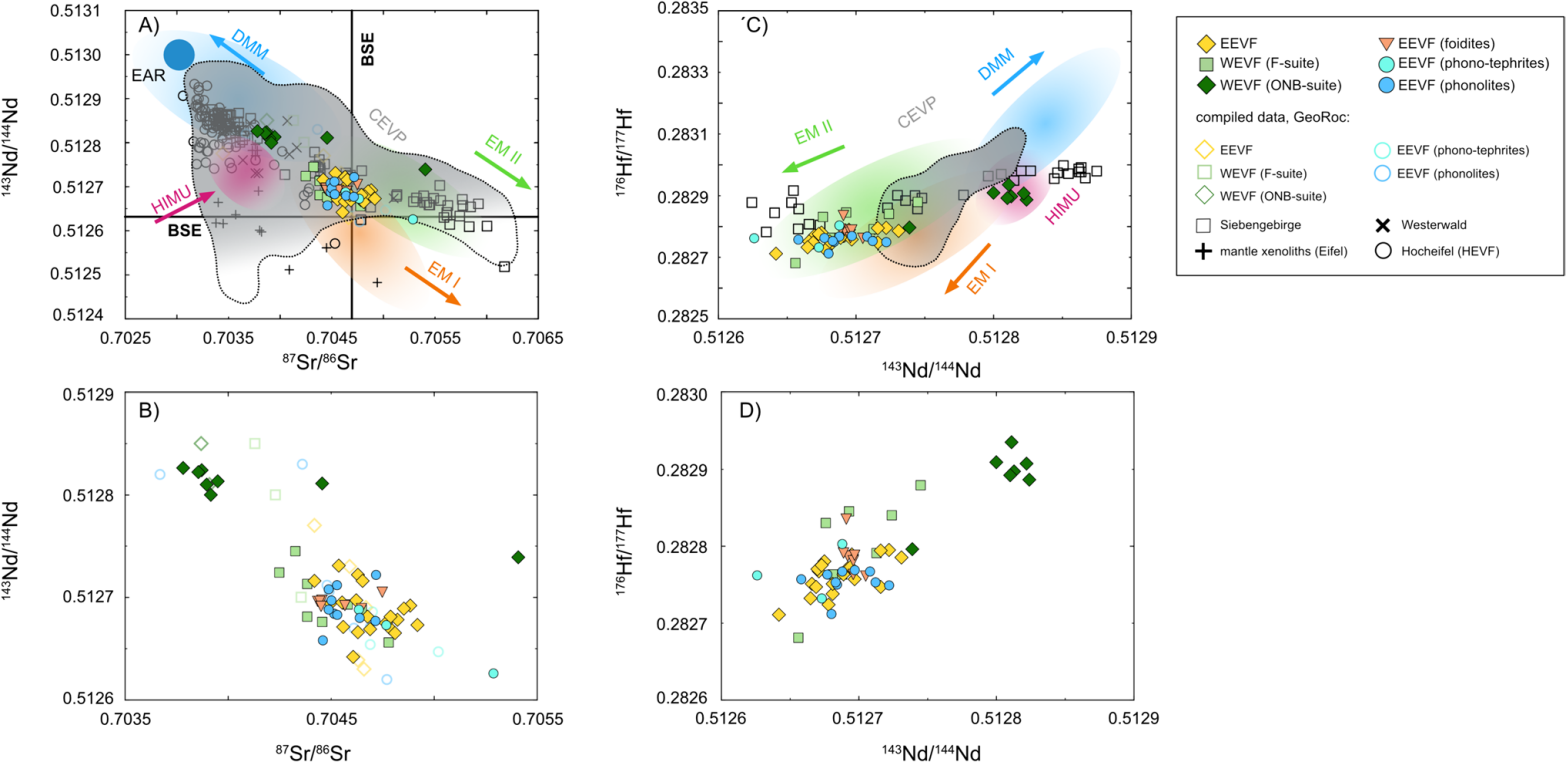
In ^143^Nd/^144^Nd vs. ^86^Sr/^87^Sr isotope space (**A**, **B**), the samples display a compositional spread, suggesting the influence of depleted mantle endmembers that are compositionally similar to DMM as well as enriched reservoirs of EM I/EM II affinity. While Sr–Nd compositions of mafic as well as differentiated lavas of both volcanic fields generally overlap, ONB-suite samples can be clearly distinguished, forming a high ^143^Nd/^144^Nd–low^86^Sr/^87^Sr group. This is further illustrated by reducing the scale of Sr and Nd isotope compositions on the axes (**B**). Additionally, two samples from the ONB-suite and one phonolite tend towards more radiogenic ^86^Sr/^87^Sr and lower ^143^Nd/^144^Nd isotope compositions, indicative of crustal contamination. In ^143^Nd/^144^Nd vs. ^176^Hf/^177^Hf isotope space (**C**, **D**), the samples display a compositional spread that is broadly similar to their respective Sr–Nd–Pb isotope compositions, suggesting the influence of a DMM/HIMU-type mantle source for the ONB-suite and an EM I-like mantle endmember for F-suite and EEVF volcanism. ONB-suite rocks can be clearly distinguished, forming a high ^143^Nd/^144^Nd– high ^176^Hf/.^177^Hf group. The compositional spread is further underlined by plotting Hf and Nd isotope compositions at higher resolution in **D**. Grey shaded areas indicate the compositional spread of CEVP volcanism, colored areas indicate compositions of classic mantle endmembers. Compositional fields and literature data have been generated using data from the GeoRoc Database (https://georoc.eu/georoc/new-start.asp)

**Fig. 5:**
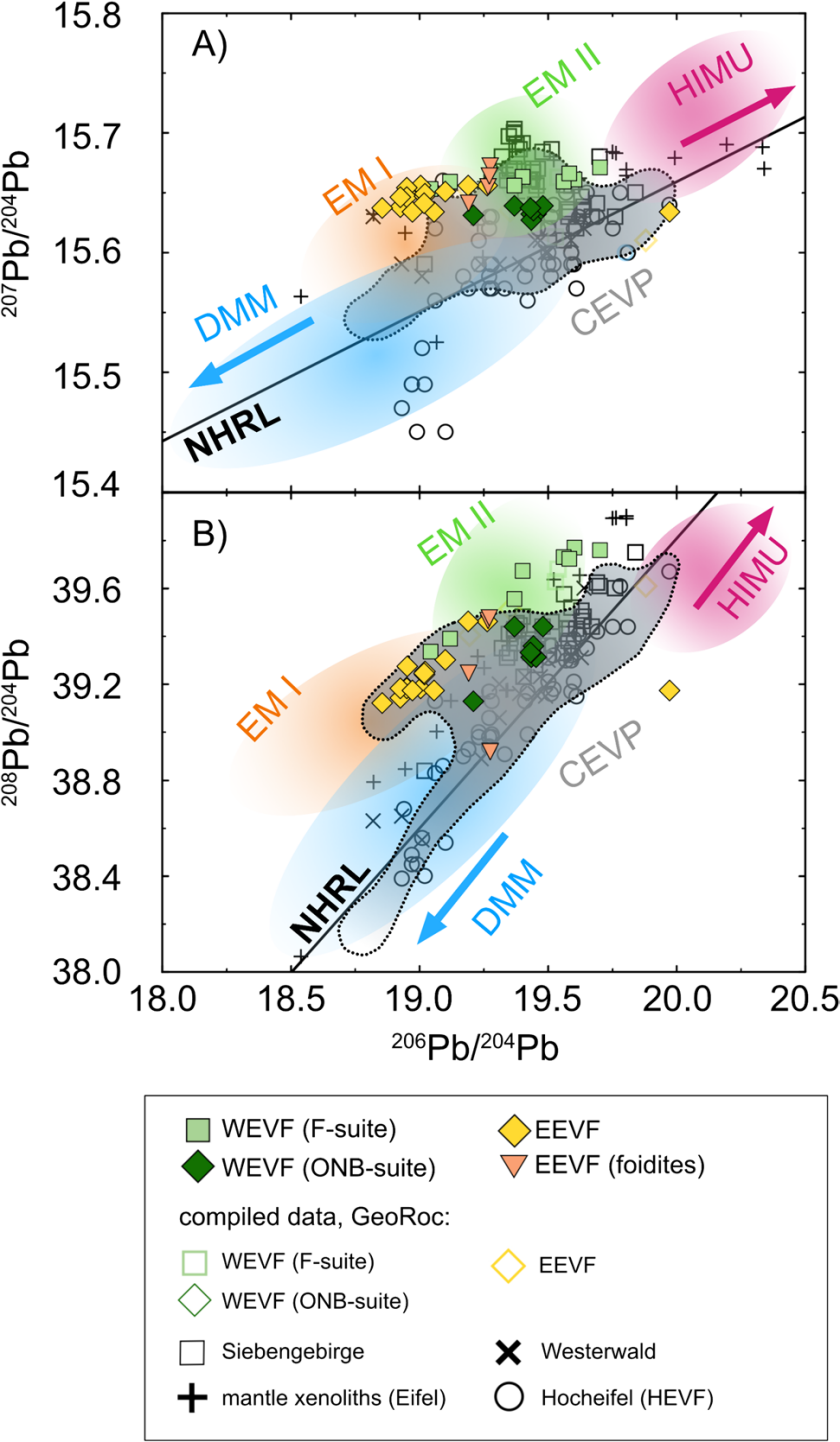
In ^207^Pb/^204^Pb ^208^Pb/^204^Pb vs. ^206^Pb/^204^Pb isotope spaces (**A**, **B**) samples of both volcanic fields plot above the North Hemispheric Reference Line (NHRL) (Hart 1984) and form a linear array. The samples display discrete compositional ranges for the ONB-, F-suite and EEVF suites. F-suite volcanic rocks trend towards generally elevated ^206^Pb/^204^Pb, ^207^Pb/^204^Pb and ^208^Pb/^204^Pb ratios and suggest a predominant influence of a HIMU-type mantle endmember, while EEVF samples display less radiogenic Pb isotope compositions similar to an EM I/DMM-like mantle reservoir. ONB-suite samples show distinctly lower ^207^Pb/^204^Pb and ^208^Pb/^204^Pb isotope compositions at given ^206^Pb/.^204^Pb ratios. Grey shaded areas indicate the compositional spread of CEVP volcanism, colored areas indicate compositions of classical mantle endmembers. Compositional fields and literature data have been generated using data from the GeoRoc Database (https://georoc.eu/georoc/new-start.asp)

Radiogenic Sr, Nd, Hf and Pb isotope compositions are listed in Table S3. Samples from the Eifel volcanic field cover a broad range of radiogenic isotope compositions with ^87^Sr/^86^Sr = 0.703855–0.705408, ^143^Nd/^144^Nd = 0.512666–0.512825; (εNd = −0.2 to +3.6), ^176^Hf/^177^Hf = 0.282681–0.282935 (εHf = −3.1 to +5.8), ^206^Pb/^204^Pb = 18.61–19.70, ^207^Pb/^204^Pb = 15.62–15.67 and ^208^Pb/^204^Pb = 38.89–39.76, respectively (Figs. 4, 5). Except for the phono-tephritic sample Ei 38 (Ettringer Bellerberg), all differentiated compositions largely overlap with the compositional spread defined by the mafic basanites and foidites (Figs. 4, 5). In isotope variation diagrams (Figs. 4, 5), samples from the Quarternary EVF plot between the compositions of DMM, HIMU, EM I, and EM II, suggesting the influence of multiple mantle endmembers. While F-suite and EEVF samples compositionally overlap at BSE-like compositions, ONB-suite samples can be clearly distinguished in ^143^Nd/^144^Nd vs. ^87^Sr/^86^Sr and ^143^Nd/^144^Nd vs. ^176^Hf/^177^Hf isotope spaces (Figs. 4, 5). The ONB samples tend towards more radiogenic ^143^Nd/^144^Nd (0.512739–0.512822) and ^176^Hf/^177^Hf isotope compositions (0.282935–0.282796) at lower ^86^Sr/^87^Sr ratios (0.703855–0.703950). However, two ONB-suite samples (Ei 58, Grube Leyendecker; Ei 28, Fächerhöhe) are shifted towards higher ^86^Sr/^87^Sr and less radiogenic ^143^Nd/^144^Nd and ^176^Hf/^177^Hf isotope compositions, suggestive of crustal contamination or mixing with F-suite source compositions. In ^206,207,208^Pb/^204^Pb isotope spaces, samples of both volcanic fields plot above the North Hemispheric Reference Line (NHRL; Hart 1984) and display discrete compositional ranges for ONB-, F-suite and EEVF samples (Fig. 5). Volcanic rocks of the F-suite tend towards elevated ^206^Pb/^204^Pb and ^208^Pb/^204^Pb ratios suggesting a predominant influence of E-MORB or HIMU-like mantle endmembers, whereas EEVF samples display less radiogenic Pb isotope compositions similar to an EM I-like mantle reservoir. In accord with Sr–Nd–Hf isotopes, ONB-suite samples also show distinct Pb isotope compositions, by having comparable ^206^Pb/^204^Pb but lower ^208^Pb/^204^Pb and ^207^Pb/^204^Pb compositions than F-suite volcanic rocks (Fig. 5). Interestingly, the foiditic rocks of the EEVF analyzed within this study are indistiguishable from the basanites and tephrites, showing overall similar Sr–Nd–Hf isotope compositions (Fig. 4). However, in ^206,207,208^Pb/^204^Pb isotope spaces, foiditic rocks compositionally link the EEVF and WEVF petrogenetic suites by displaying intermediate Pb isotope compositions compared to both volcanic fields and the WEVF petrogenetic suites (Fig. 5).

### ^187^Re–^187^Os isotope and HSE compositions

For ^187^Re–^187^Os and HSE analysis we have selected 13 representative samples from the EEVF, the F-suite and the ONB-suite, respectively. In a primitive mantle-normalized HSE diagram (Fig. S5) the samples analyzed within this study display typical enrichments of the PPGE’s (Pd-subgroup, Rh-Pt–Pd) compared to the IPGE’s (Ir-subgroup, Os-Ir-Ru), which is characteristic for asthenospheric mantle melts (e.g., Mondal 2011). Measured Re/Os ratios reveal a large range from 4.22 to 72.1, which is equivalent to ^187^Re/^188^Os ratios ranging from 21.1 to 356 (Table S2). Similarly, variable patterns are found for Os concentrations (2–148 ppt) and ^187^Os/^188^Os ratios that vary from near primitive upper mantle values (0.139, primitive upper mantle = 0.129; Meisel et al. 1996), to ratios as radiogenic as ^187^Os/^188^Os = 0.697 which are still substantially lower compared to ratios found with the upper-continental crust. Further, the samples analyzed in this study display co-variations of ^187^Os/^188^Os and Re/Os ratios (Fig. 11).

In general, measured trace element and Sr–Nd–Hf–Pb isotope compositions of our samples from the Quaternary EVF are in good agreement with previously published data for the Quaternary and Tertiary Eifel volcanic fields (e.g., Wörner et al. 1986; Jung et al. 2006; Fekiacova et al. 2007) as well as for the CEVP (e.g., Jung and Masberg 1998; Jung and Hoernes 2000; Jung et al. 2006, 2011; Fekiacova et al. 2007; Lustrino and Wilson 2007; Lustrino 2011; Pfänder et al. 2012, 2018). The measured Os isotope compositions of the quaternary Eifel samples are similar to published age corrected data for Tertiary volcanic rocks from the CEVP (e.g., Jung et al. 2005, 2011; Mayer et al. 2013, 2018) that also revealed radiogenic Os isotope compositions above the mantle value.

## Discussion

### Effects of fractional crystallization

Positive co-variations of CaO, Fe_2_O_3_, Ni and Cr and a negative co-variation of Al_2_O_3_ contents with MgO (Fig. S1) confirm typical magmatic differentiation trends, caused by predominant fractionation of olivine and clinopyroxene. More differentiated melts (including the phono-tephrite and phonolite groups) also show trends towards generally lower Ce/Pb and Nb/U ratios (Fig. S2). This is likely an indication for fractional crystallization. In detail, titanite is responsible for the selective fractionation of Nb and Ta, garnet for Zr and Hf (e.g., Green et al. 2000; Klemme et al. 2002; Tiepolo et al. 2002), apatite for MREE (Fujimaki 1986; Prowatke and Klemme 2006), and sanidine for Ba and Pb (e.g., Viereck 1984). Further, the lack of negative Eu anomalies (Fig. 3) in more differentiated melts could provide evidence for the absence of large-scale plagioclase fractionation as suggested by plagioclase bearing cumulate xenoliths that have been reported from the area (Loock et al. 1990; Viereck 1984). However, more detailed investigations of differentiated rocks from the quaternary EVF that have been sampled at the Laacher See, and Wehr- volcanic complexes have provided evidence for plagioclase fractionation taking place at upper-crustal depths (Schmincke et al. 1983; Viereck 1984; Wörner and Schmincke 1984a, b; Schmincke 2007). In particular the presence of green-core clinopyroxene has suggested polybaric differentiation of magmas during their ascent (Duda and Schmincke 1985). Following previous work (e.g., Viereck 1984; Mertes & Schmincke 1985), the compositions of the samples analyzed within this study are in good agreement with enhanced fractional crystallization of olivine, pyroxene, titanite, nepheline, sanidine and leucite, and a rather limited influence of crustal contamination for mafic samples (Fig. S2). The effects of crustal contamination on the compositions of differentiated samples are further assessed below using Sr isotopes.

### Effects of crustal assimilation

With respect to identifying mantle sources, data for continental intraplate basalts require a more careful assessment due to the potential effects of crustal assimilation and metasomatism of the lithospheric mantle. Given that continental intraplate volcanic rocks erupt through variably thick, compositionally heterogeneous lithosphere, diverse crustal materials might be entrained by AFC processes (DePaolo 1981). With a focus on the CEVP, previous studies have already largely precluded a significant role of crustal contamination by comparing the geochemical composition of mafic and differentiated lavas (e.g., Wörner et al. 1986; Jung et al. 2006; Fekiacova et al. 2007; Kolb et al. 2012; Jung et al. 2012; Schneider et al. 2016; Mayer et al. 2018). Despite these results, other, more sophisticated approaches that focused on energy-constrained AFC-modelling (Bohrson and Spera 2001; Spera and Bohrson 2001) have revealed that the assimilation of lower-crustal granulites might have played a significant role for more differentiated lavas (e.g., Jung et al. 2006; Kolb et al. 2012; Schneider et al. 2016). This was also postulated for Vogelsberg tholeiites based on Os isotopes (Jung et al. 2011).

**Fig. 6:**
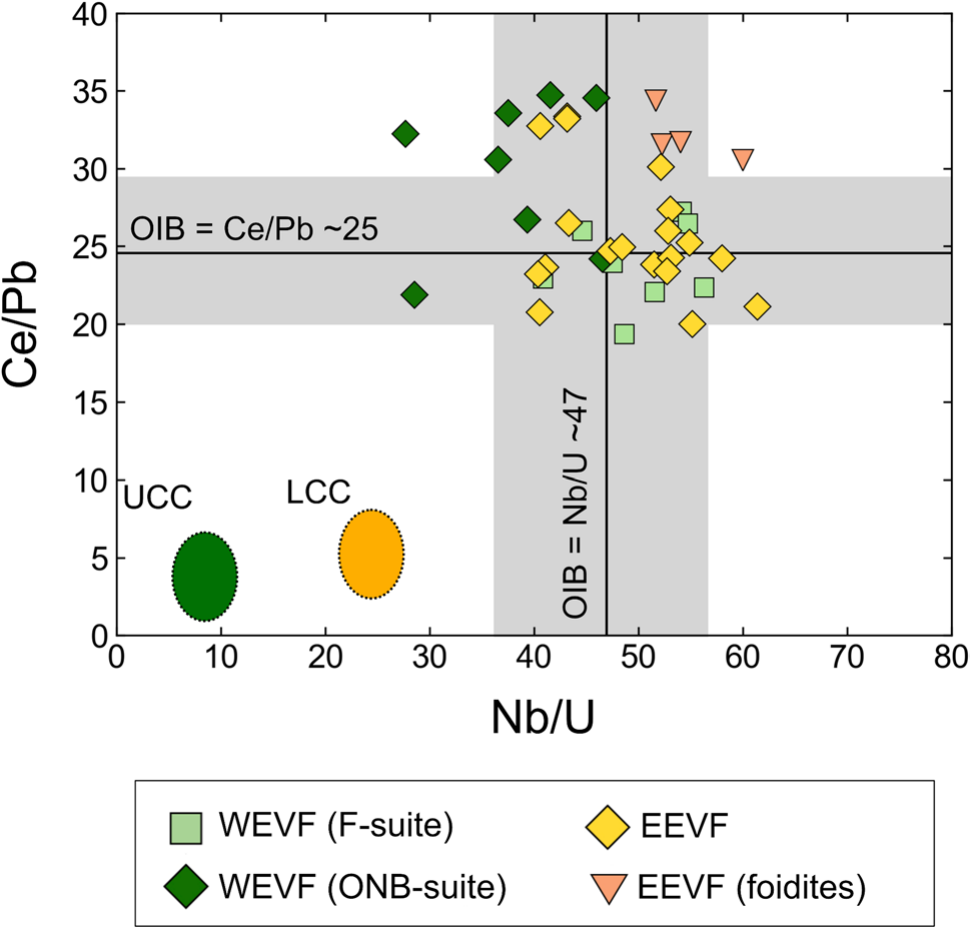
Measured Ce/Pb and Nb/U ratios of mafic samples generally agree with values of OIBs but exceed the canonical ratios of ~25 Ce/Pb and ~47 Nb/U that have been inferred for OIBs (Hofmann et al. 1986)

Considering the samples analyzed within this study, the low SiO_2_ (<50 wt.%), high MgO (>6.5 wt.%) and high Ni and Cr concentrations within mafic lavas from the EVF generally attest to their primitive nature. This is also in good agreement with the presence of ultramafic mantle xenoliths that occur in mafic lavas of the WEVF (Witt-Eickschen et al. 2003; Schmincke 2007). For typical canonical trace element ratios such as Ce/Pb and Nb/U (Hofmann et al. 1986; Hofmann 2003), the majority of our samples plot within the compositional field defined by OIBs (Fig. 6) and, therefore, do not show significant evidence for crustal contamination. Likewise, there are no clear co-variations of radiogenic isotope signatures with MgO (Supplemental Fig. 3).

**Fig. 7:**
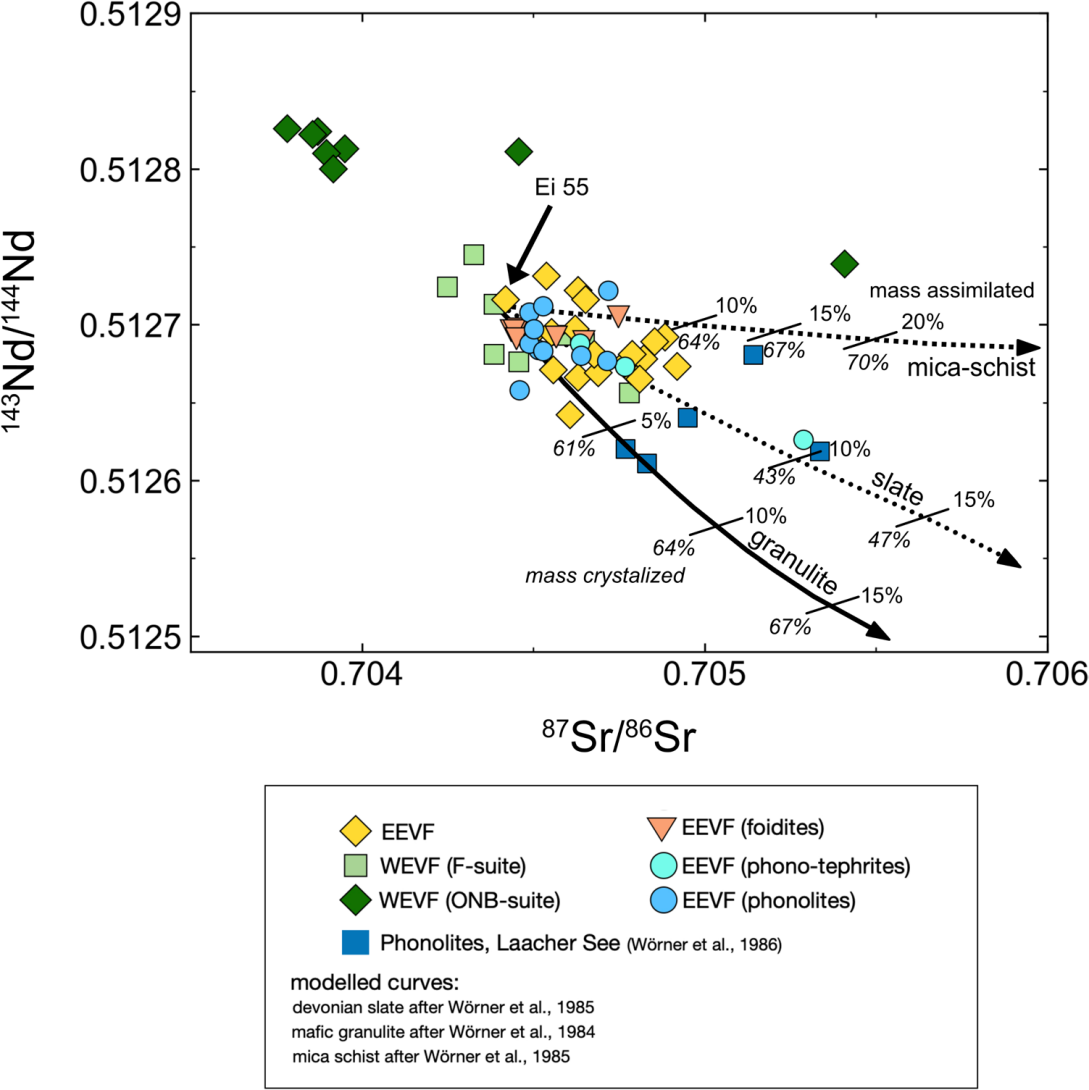
Measured compositions of EVF samples in comparison to energy-constrained assimilation-fractional-crystallization (EC-AFC) models after Bohrson and Spera (2001) and Spera and Bohrson (2001), using modelling parameters given in Schneider et al. (2016). Sr–Nd isotope modelling of mafic as well as differentiated rocks (compositions of typical crustal crocks found within the CEVP; Wörner et al. 1985, 1986) reveals that the amounts of assimilated material cannot have exceeded 3–6% at 40–60% fractional crystallization. However, one phono-tephrite sample (Ei 38) displays radiogenic Sr isotope compositions that are consistent with over to 10% assimilated material. The low ratio of assimilation/fractional crystallization (0.08–0.3) is consistent with similar values of Bohrson and Spera (2001)

Given the limited occurrence of differentiated volcanic rocks within the WEVF (Schmincke et al. 1983; Mertes and Schmincke 1985) and the apparent age difference between F-suite, EEVF and ONB-suite volcanism, we have focused our assessment of crustal contamination on differentiated lavas from the Rieden Subfield of the EEVF for which we report new data here. To estimate the potential effects of crustal contamination on the composition of EEVF samples more quantitatively (using Rieden and previously published Laacher See data), we have carried out a simple energy-constrained assimilation-fractional-crystallization (EC-AFC) model by Bohrson and Spera (2001) and Spera and Bohrson (2001), with a particular focus on Sr–Nd isotopes. As initial composition, we have used the MgO-rich basanite Ei 55 (Bräuning volcano) that represents a mafic eruptive center, located within the Rieden volcanic complex. For the EC-AFC model at various depths we have used modelling parameters that were previously used for the nearby Siebengebirge volcanic field (Schneider et al. 2016). As representative for the lower crustal contaminant, we used granulite sample S32 from Stosch and Lugmair (1984), as representative for an upper crustal contaminant a Devonian slate (S200) as well as a mica-schist (S144) from Wörner et al. (1985). All these rocks have been previously investigated for their Sr–Nd isotope compositions and can be regarded as compositionally representative of the various crustal levels underneath the EVF (Wörner et al. 1985). The results of the modelling calculations are illustrated in Fig. 7.

Applying the EC-AFC model to the mafic and differentiated lavas analyzed within this study shows that their Sr–Nd compositions overlap with the modelled assimilation curves for all crustal endmembers (Fig. 7). However, given that all of the potential assimilants are characterized by substantially more radiogenic ^87^Sr/^86^Sr and unradiogenic ^143^Nd^144^Nd compositions than in our samples from the EEVF, potential crustal assimilation must have been rather insignificant, even for the phonolites. Further, regardless of which assimilant is assumed, the Sr–Nd isotope composition of the mafic as well as of the differentiated rocks only permit a maximum of 3–6% assimilated material at 40–60% fractional crystallization. However, one phono-tephrite (Ei 38, Ettringer Bellerberg) exhibits significantly more radiogenic Sr isotope compositions (^87^Sr/^86^Sr = 0.705288) that could be consistent with over 10% assimilated material (Fig. 7), or alternatively with a different type of assimilant. Likewise, two basanites from the ONB-suite (Ei 58a and Ei 28) which display more radiongenic ^87^Sr/^86^Sr compositions (0.705409 (Ei 58a) and 0.704457 (Ei 28) in comparison to other ONB samples (Fig. 7), also indicating some crustal assimilation of these two samples.

For all other phonolites analyzed, the Sr–Nd isotope compositions seem to be rather unaffected by AFC processes (Figs. 4, 7), which is likely because the Sr and Nd inventories of these lavas are buffered against crustal assimilation due to their high element concentrations. In agreement with such a view, Sr isotope compositions in phonolites from the Laacher See eruption are much more radiogenic in their Sr isotope compositions than all phonolites analyzed here (Wörner et al. 1985), because these phonolites underwent prior plagioclase fractionation which lowered their Sr contents.

### Mantle source characteristics

#### Melting conditions

Given the limited influence of crustal assimilation on the compositions of mafic volcanic rocks from the EVF analyzed here, it can be inferred that their radiogenic isotope compositions reflect the composition of their mantle source reservoirs. In this regard, several studies have suggested the presence of one or several small mantle plumes underneath the CEVP (e.g., Granet et al. 1995; Goes et al. 1999, 2000; Ritter et al. 2001; Ritter and Christensen 2007). However, it has been argued that the expected diameter (~2000 km) of a single mantle-plume rising upwards from the core-mantle boundary is inconsistent with the dimensions of the individual CEVP eruptive centers (Wilson and Downes 1991; Fekiacova et al. 2007). Moreover, seismological investigations have indicated P-wave anomalies that more are consistent with the presence of small-scale “plumelets” rooted in the mantle-transition zone rather than in the lower-mantle (Ritter et al. 2001; Ritter and Christensen 2007). Several studies have therefore suggested that CEVP volcanic rocks are rather sourced from the upper-asthenospheric and lithospheric mantle, the latter being refertilized as a consequence of enhanced subduction recycling during the Variscan orogeny (e.g., Wilson and Downes 1991; Witt-Eickschen et al. 2003). While previous studies on mantle xenoliths found within EVF lavas have indicated that the upper-mantle underneath the CEVP mostly consists of metasomatized spinel peridotite (e.g., Witt-Eickschen and Kramm 1998; Witt-Eickschen et al. 2003), other studies have provided evidence for the involvement of residual garnet in the source of CEVP volcanism and mixtures of partial melts generated in the spinel- as well as the garnet stability field (e.g., Haase et al. 2004; Jung et al. 2006; Fekiacova et al. 2007; Mayer et al. 2018).

**Fig. 8:**
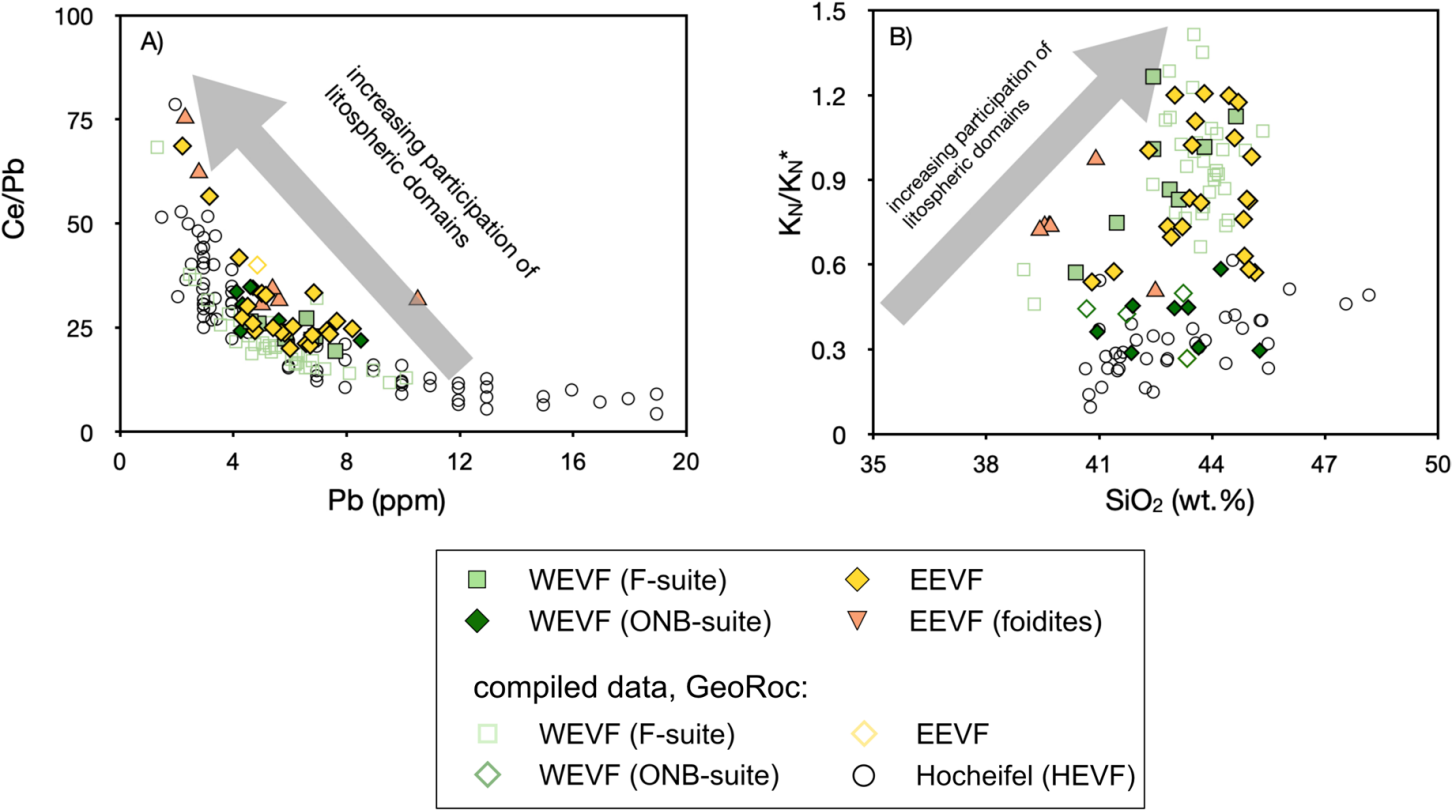
In Ce/Pb vs. Pb (**A**) and K_N_*/K_N_ vs. SiO_2_ (**B**) space, samples of both volcanic fields display a negative co-variation of Ce/Pb vs. Pb (**A**) and a positive co-variation of K_N_*/K_N_/ vs. SiO_2_ (**B**). This indicates that these trace element ratios are fractionated during partial melting (e.g., Mayer et al. 2018). These features are best explained by variable degrees of partial melting in the presence of amphibole or phlogopite (Mayer et al. 2018)

Considering the samples analyzed in this study, plots of Ce/Pb vs. Pb and K_N_*/K_N_ vs. SiO_2_ (Fig. 8) provide evidence for residual mineral phases such as amphibole and phlogopite in the source of EVF volcanism. Given that ONB-, F-suite (WEVF) and EEVF samples display distinct ranges of negative K and Pb anomalies in primitive mantle-normalized trace-element diagrams, a co-variation of Ce/Pb vs. Pb (Fig. 11) indicates that these trace element ratios are fractionated during partial melting. This feature was also previously observed in other volcanic rocks of the CEVP (e.g., Jung and Hoernes 2000; Kolb et al. 2012; Mayer et al. 2018) and is best explained by variable degrees of partial melting in the presence of residual amphibole or phlogopite. High Ce/Pb and low K_N_/K_N_* therefore might indicate small degrees of partial melting whereas low Ce/Pb and high K_N_/K_N_* together with higher SiO_2_ contents might result from the release of Pb and K during amphibole or phlogopite breakdown at higher degrees of partial melting and shallower depths (e.g., Jung and Hoernes 2000). In good agreement with previous studies, our data indicate that ONB- as well as F- and EVF are sourced from mantle sources that have been affected by mantle metasomatism involving the formation of secondary phlogopite and amphibole (e.g., Mertes and Schmincke 1985; Witt-Eickschen and Kramm 1998; Witt-Eickschen et al. 2003).

**Fig. 9:**
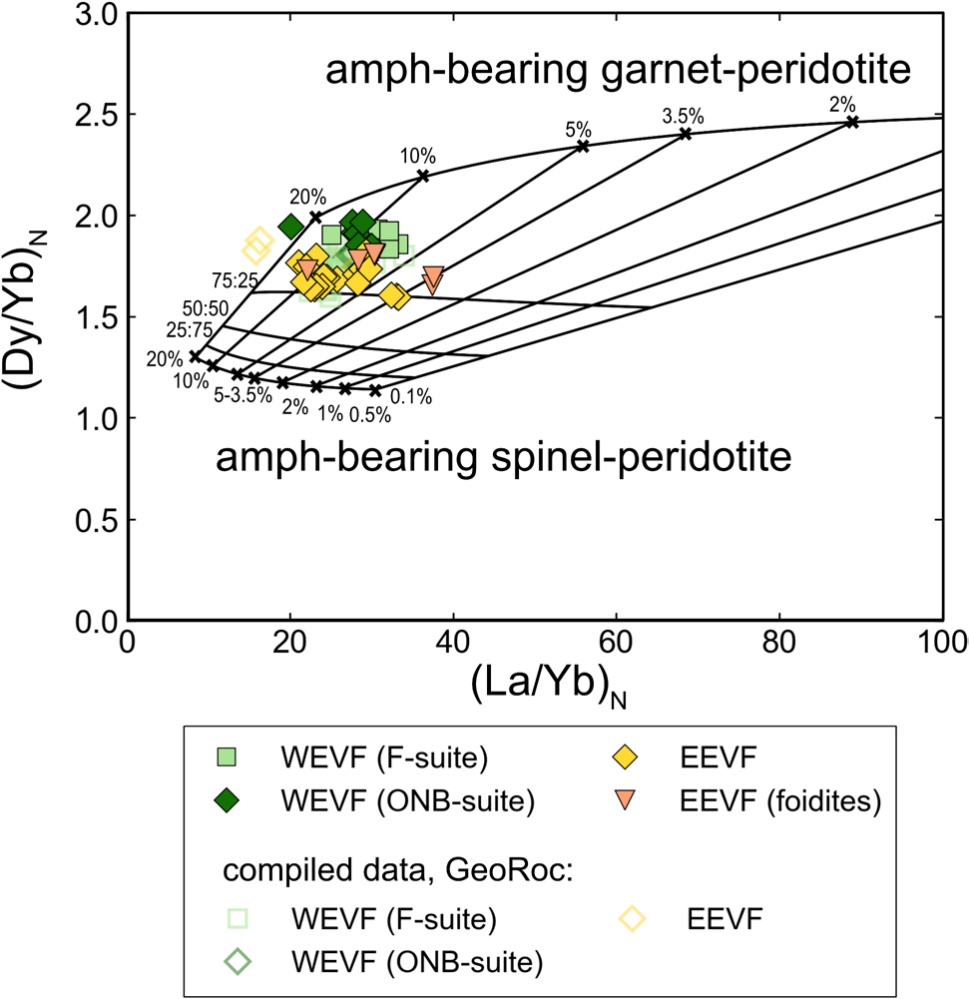
In a plot of (Dy/Yb)_N_ vs. (La/Yb)_N_, our data are in good agreement with mixing of partial melts originating within the spinel- as well as the garnet stability field. Therefore, our data provide strong evidence for mixing of melts originating from variable depths within the mantle (e.g., Haase et al. 2004; Jung et al. 2006; Fekiacova et al. 2007; Lustrino and Wilson 2007; Mayer et al. 2018). The model has been created using the PetroGram workbook program of Gündüz and Asan (2021) and the parameters, source compositions and mineral proportions are after Jung et al. (2006) and references therein

To evaluate the melting conditions, several models carried out for CEVP volcanism have focused on REE systematics, such as plots of Dy/Yb vs. La/Yb (e.g., Haase et al. 2004; Jung et al. 2006; Mayer et al. 2018; Jung et al. 2006), as well as SiO_2_ contents and major element ratios such as CaO/Al_2_O_3_ (e.g., Fekiacova et al. 2007). Given that the HREE are strongly fractionated by garnet, for near primitive melts a plot of primitive mantle normalized (Dy/Yb)_N_ vs. (La/Yb)_N_ can be used to distinguish between partial melting within the garnet peridotite and the spinel-peridotite stability fields (e.g., Thirlwall et al. 1994; Jung et al. 2006). If pooling of melts sourced from spinel- and garnet peridotite occurs, the samples investigated should plot along linear mixing arrays between both sources (e.g., Jung et al. 2006). In Fig. 9 non-modal batch-melting curves of a garnet- amphibole peridotite and a spinel-amphibole peridotite are shown that were calculated using the PetroGram workbook program of Gündüz and Asan (2021) and the parameters, source compositions and mineral proportions of Jung et al. (2006 and references therein). The calculated model curves in Fig. 9 imply that mafic Quaternary Eifel magmas, plotting in a range from (Dy/Yb)_N_ = 1.59–1.96 and (La/Yb)_N_ = 20.1–37.6, could result from 3 to 15% partial melting. The data further indicate that partial melts from garnet-bearing sources were mixed with melts from spinel-bearing sources (Fig. 9). Mafic samples from the WEVF (ONB- and F-suite) trend towards more elevated (Dy/Yb)_N_ (1.85–1.96), suggesting a greater contribution from garnet-bearing sources compared to samples from the EEVF (Fig. 9). Further, some Mg-rich foiditic samples from the EEVF display elevated (La/Yb)_N_, suggesting generally lower degrees of partial melting compared to the basanites (Fig. 9). This is in good agreement with relatively low CaO/Al_2_O_3_ ratios at low SiO_2_, suggesting increasing degrees of partial melting from foidites to basanites as well as confirming the influence of residual garnet in the source of the WEVF volcanic rocks (Fig. 9; Fig. S6) (e.g., Jung et al. 2006; Kolb et al. 2012).

Collectively, our data provide strong evidence for a greater influence of deeper, garnet-bearing mantle sources for West Eifel ONB- and F-suite volcanism, whereas samples from the EEVF might be sourced from shallower depths in the spinel-peridotite field. Our data are in good agreement with experimental results that have indicated basanites to be generated from peridotite sources by melting degrees over 1% (Kushiro 1996). Following the major and trace element-based modelling approaches that have previously been carried out for tertiary CEVP volcanism, a temperature and pressure range of 1250–1300 °C and 25–30 kbar, respectively, can be deduced, corresponding to depths of ca. 75–90 km, along the garnet-spinel stability transition zone (e.g., Mertes and Schmincke 1985; Kushiro 1996; Fekiacova et al. 2007).

#### Nature of the mantle sources involved in Quaternary Eifel volcanism

**Fig. 10:**
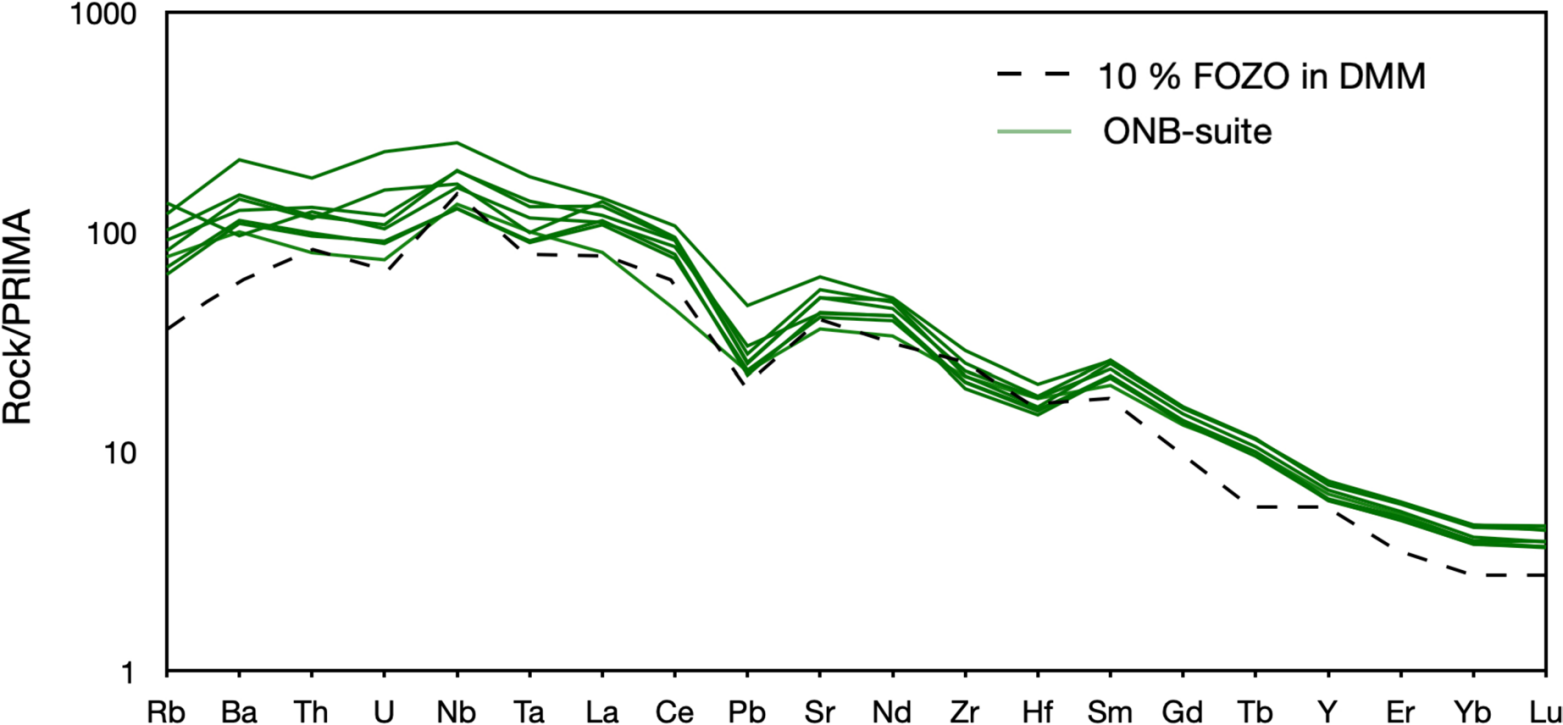
Modeling of mixing between a FOZO- and a DMM-like melt to create enriched hybrid melts as found in the ONB suite. The batch melting approach is adopted from Shaw (1970) and modelling parameters from Fekiacova et al. (2007) are used. Using the simple modal batch melting approach our results indicate that an enriched hybrid melt, created by mixing of 10–14% of FOZO-type melt (Raivavae lavas; RVV 360A; Lassiter et al. 2003) and 86–90% of a DMM melt perfectly resembles the trace-element patters as found for our ONB-type lavas

In general, radiogenic isotope data of the samples investigated here overlap data reported in previous studies on volcanism of the Eifel and neighboring volcanic fields (e.g., Siebengebirge, Westerwald, Hocheifel) (e.g., Mertes and Schmincke 1985; Fekiacova et al. 2007; Jung et al. 2006, Haase et al. 2004; Kolb et al. 2012) but now enable to discriminate more compositional groups (Figs. 4, 5). In Sr–Nd–Hf–Pb isotope spaces (Figs. 4, 5) the samples exhibit a large variation that implies a substantial heterogeneity of the mantle sources involved. Samples of the ONB-suite form a discrete compositional group with low ^87^Sr/^86^Sr, high ^143^Nd/^144^Nd and high ^176^Hf/^177^Hf (Fig. 4). ONB-suite samples thus display DMM/FOZO-like compositions, suggesting a major-influence of a FOZO-like reservoir on young volcanism in the WEVF (Figs. 4, 10). The chemical compositions of ONB-suite samples further overlap with compositions of lavas from many other Mid European Tertiary volcanic fields such as Hocheifel and Westerwald. In contrast, samples from the F-suite and the EEVF plot close to the bulk silicate earth (BSE) values in Sr–Nd–Hf isotope spaces, suggesting the influence of an EM I-like mantle endmember (Fig. 4). In Pb-Pb isotope spaces, samples of the F-suite and the EEVF form a linear trend that is consistent with mixing of source reservoirs having variable time-integrated U/Pb and Th/Pb ratios. While samples from the F-suite display more radiogenic ^206^Pb/^204^Pb isotope compositions (19.1–19.6) that are also in good agreement with an enhanced contribution of a HIMU-like endmember, samples from the EEVF appear to be sourced from a reservoir with lower U/Pb and Th/Pb that is compositionally similar to the EM I-like mantle endmember. In contrast, ONB-suite samples display generally lower ^207^Pb/^204^Pb and ^208^Pb/^204^Pb at given ^206^Pb/^204^Pb ratios and thus form a separate trend, roughly parallel to the trend defined by F-suite and EEVF volcanism (Fig. 5). Moreover, ONB-suite samples are compositionally more similar to previously analyzed samples from the Tertiary HEVF (e.g., Jung et al. 2006; Fekiacova et al. 2007) in Pb-Pb isotope space. In contrast to the analyzed samples from the EEVF and the F-suite, that resemble a EM I–HIMU mixing line, the sub-parallel array of the ONB suite samples provides further evidence for a unique source to the ONB-type volcanism that might be best resembled by a FOZO-type mantle source (Figs. 4, 5, 10). The overall heterogeneous isotope composition thus indicates the participation of at least three different mantle endmembers. Hence, Quaternary volcanism in the EVF cannot be explained by simple partial melting of a single plume source alone. Instead, our data are in good agreement with previous models, suggesting that CEVP volcanism is sourced from a FOZO-type component that is located within the asthenosphere (e.g., Jung et al. 2006; Fekiacova et al. 2007) and provides a source for Na_2_O-rich basalts (ONB-suite), as well as more enriched lithospheric sources of EM I and potentially HIMU affinities that might be located close to the thermal boundary layer, providing the sources for K_2_O-rich (F-suite and EEVF) basalts (Wilson and Downes 1991; Jung et al. 2006).

Given the unique character of ONB-suite volcanic rocks, several lines of evidence (high Mg#, Ni and Cr) suggest that volcanism younger than 80 ka represents the most mafic endmember with a more pronounced asthenospheric origin of the primary melts, which is also suggested by our melting model, where for the ONB-suite a larger proportion of melts originated from the garnet peridotite field (Fig. 9; Fig. S4). Given the overall geochemical similarity of Quaternary ONB-suite and Tertiary HEVF rocks with compositions reported from a FOZO-type source, we have therefore adapted previous isotope and trace-element modelling constraints for the Hocheifel volcanic field (Fekiacova et al. 2007). Using a batch melting approach (Shaw 1970) we have calculated mixing of a FOZO-type melt (represented here by Raivavae lava RVV 360A in Lassiter et al. (2003)) and a DMM-melt (Salters and Stracke 2004) (Fig. 10), indicating that mixing of 10–14% of FOZO-type melts and up to 90% of DMM melts, can account for compositions of the ONB-suite as well as the HEVF suite (Fekiacova et al. 2007). However, as already noted in the previous section, all EVF samples analyzed within this study display evidence from negative K and Pb anomalies for the involvement of residual amphibole or phlogopite. Both minerals are stable within the lithosphere or at the thermal boundary layer (TBL) but may also occur at larger depths (Condamine et al. 2016; Sokol et al. 2017). Hence, our results are in good agreement with previous models claiming that mafic CEVP melts were generated by partial melting close to the TBL (e.g., Wilson et al. 1995), but contributions from deeper asthenospheric mantle sources are also possible.

Given the distinct compositional differences of Quaternary volcanic rocks from the EVF, new insights on their mantle sources can now be retrieved from ^187^Re–^187^Os isotope systematics. Samples from the EVF analyzed within this study are the first Quaternary lavas analyzed for ^187^Re–^187^Os isotopes from the CEVP. Compared to the mantle value, they generally display elevated ^187^Os/^188^Os ratios in a range from 0.140 to 0.699 (Table S4). This range is in good agreement with previously reported, age corrected compositions of Tertiary intraplate volcanic rocks from the CEVP that yielded ^187^Os/^188^Os ratios up to 0.750. (Blusztajn and Hegner 2002; Jung et al. 2005, 2011). The elevated ^187^Os/^188^Os compositions (0.140–0.699) as observed in Eifel volcanic rocks from this study further argue against non-metasomatized mantle sources which should have slightly sub- to near-chondritic ^187^Os/^188^Os ratios of ≤0.127 (Shirey and Walker 1998). As this affects both ONB as well as F-suite and EEVF samples, the Os isotope compositions further underline that none of the Quaternary volcanic suites in the EVF are direct derivatives of a rising mantle plume and that all sources have been affected by mantle metasomatism.

**Fig. 11:**
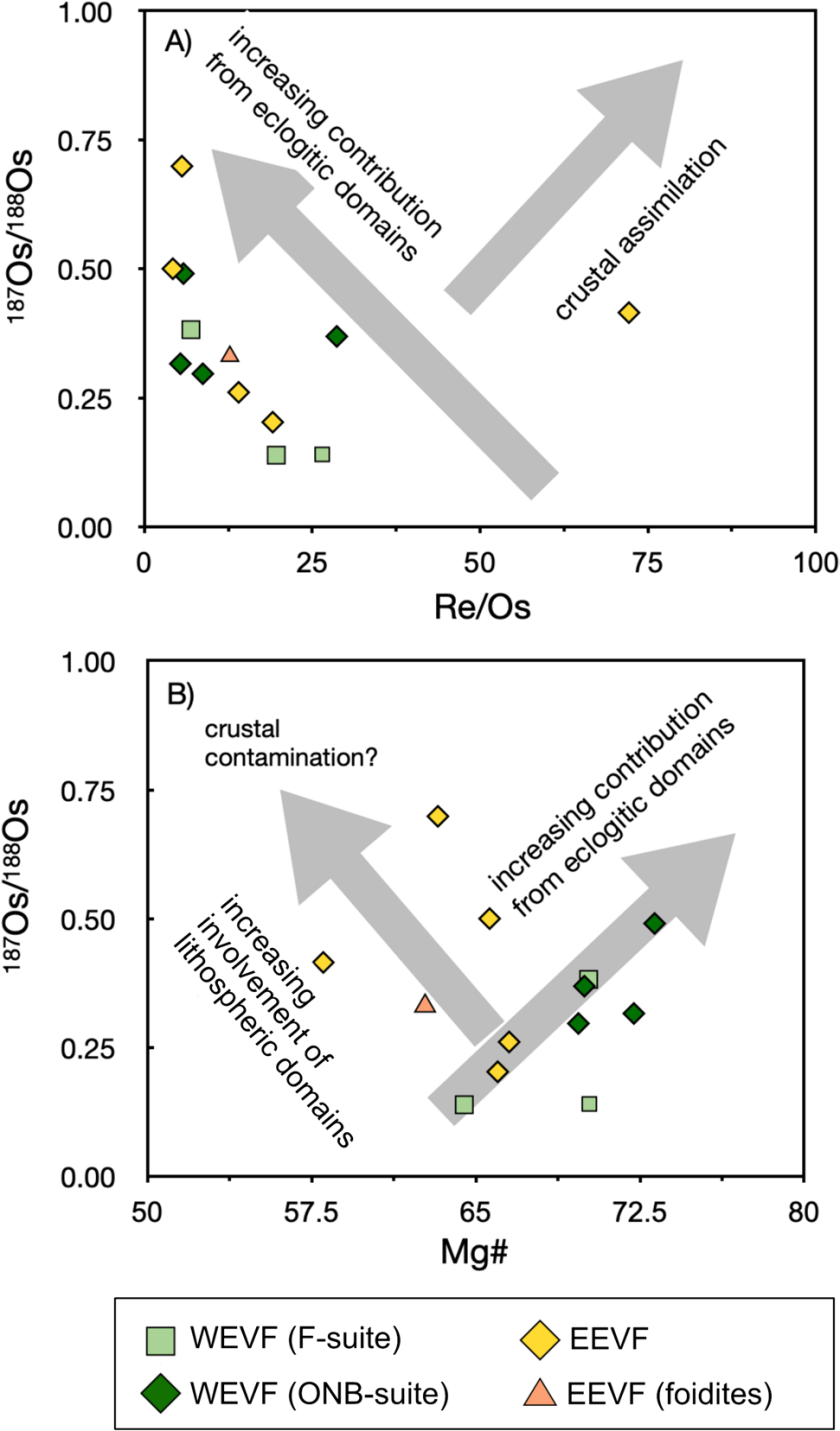
In ^187^Os/^188^Os vs. Re/Os (**A**) and ^187^Os/^188^Os vs. Mg# (**B**) space, most samples display co-variations. Generally lower Re/Os ratios at increasing ^187^Os/^188^Os isotope compositions (**A**) and a tentative positive co-variation of ^187^Os/^188^Os and Mg# in WEVF lavas are in good agreement with the influence of residual eclogites within the source of EVF volcanism. However, direct melting of eclogite produces melts with generally lower MgO concentrations that do not agree with the increasing ^187^Os/^188^Os at increasing Mg# for WEVF lavas (**B**). As such, ONB-suite rocks cannot represent direct melts of an eclogitic source and rather represent mixed partial melts of an eclogitic source and lithospheric-mantle melts

Given that Re behaves highly incompatible during partial melting (Shirey and Walker 1998; Hauri 2002), the continental crust is characterized by high Re/Os that evolves towards highly radiogenic ^187^Os/^188^Os compositions over time (>1.5) (Asmerom and Walker 1998). Hence, crustal contamination may also affect ^187^Os/^188^Os, however, Os contents in continental crust are usually very low and magmas are insignificantly affected at low degrees of crustal assimilation. Indeed, substantially elevated ^187^Os/^188^Os compositions in mafic and compositionally primary volcanic rocks from the CEVP (nephelinites, basanites, and tholeiites) have previously been inferred to reflect crustal contamination (Blusztajn and Hegner 2002; Jung et al. 2005, 2011). However, while we have already excluded a substantial influence of crustal contamination, an increase of ^187^Os/^188^Os with decreasing Re/Os in the EVF samples analyzed (Fig. 11A) further indicate that the influence of a Re-enriched crust must have been insignificant (Hauri 2002).

#### Evidence for residual, carbonated eclogites in the source of the Eifel volcanic fields

**Fig. 12:**
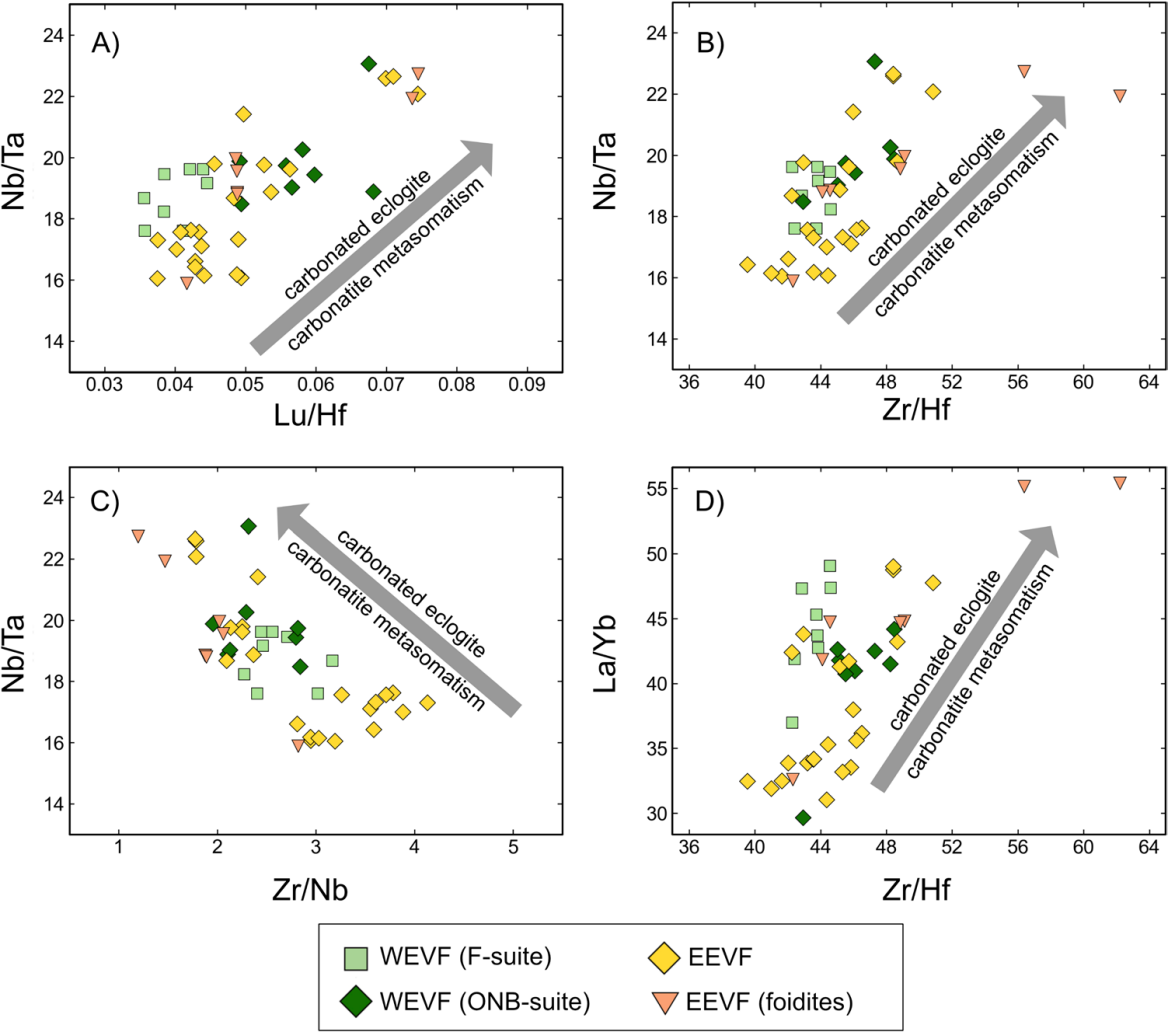
Plots of Nb/Ta vs. Lu/Hf- (**A**), Nb/Ta vs. Zr/Hf- (**B**), Nb/Ta vs. Zr/Nb- (**C**) and La/Yb vs. Zr/Hf (**D**). Ratios of Nb/Ta exceed compositions reported for OIBs (Nb/Ta = ~15–16 (Pfänder et al. 2012) and are positively correlated with Lu/Hf (**A**) and Zr/Hf (**B**) and negatively correlated with Zr/Nb (**C**) ratios. Similar to a previous study on Vogelsberg lavas, our data thus suggest the influence of a reservoir that has been subjected to carbonatite metasomatism (Pfänder et al. 2012). This carbonatite metasomatism might derive from carbonated eclogite (e.g., Dasgupta et al. 2004). Further, WEVF ONB-suite and EEVF foidites generally display elevated Zr/Hf and La/Yb (**D**). This is similar to what has been observed for volcanic rocks from the Siebengebirge and indicates element fractionation through Ca-rich almandine-bearing residual carbonated eclogites (Kolb et al. 2012)

The co-variation between ^187^Os/^188^Os and Re/Os rather suggests a more complex, multi-stage process involving residual eclogites. With respect to lithospheric mantle components underneath the CEVP, previous studies on mantle xenoliths from the EVF have reported ^187^Os/^188^Os ratios ranging from 0.114 to 0.142, indicating that the Os isotope composition of some of these xenoliths have also been overprinted by mantle metasomatism (Schmidt and Snow 2002; Fischer-Gödde et al. 2011). Notably, a previous study by Kolb et al. (2012) has already proposed the influence of residual eclogites in the source of CEVP volcanism. Given that residual eclogites tend to evolve towards highly radiogenic ^187^Os/^188^Os ratios due to elevated Re/Os ratios of their precursor rocks, we therefore argue that the observed Re–Os isotope features in EVF rock are in accord with recycled mafic crustal materials. Additionally, partial re-melting of such components will result in the release of Os, but not of Re that is strongly sequestered into garnets (Righter and Hauri 1998; Hauri 2002). Residual eclogites could thus explain the co-variation between ^187^Os/^188^Os and Re/Os and the increase for ^187^Os/^188^Os with increasing Mg# ratios for WEVF lavas (Fig. 11). Given that an increasing ^187^Os/^188^Os and Mg# is best represented by ONB-suite volcanic rocks, our data do thus tentatively suggest a major influence of residual eclogites within the sources of younger, WEVF volcanism. This is further corroborated by the elevated Zr/Hf (Fig. 12). Unfortunately, it is difficult to quantify the amount of residual eclogite required, as trace element concentrations and isotope compositions (in particular of Os) and mineral modes are unknown. However, previous modeling for ocean island basalts using high precision HSFE data (not available for the EVF) suggest that only a few percent of residual eclogite may be required (Pfänder et al. 2007).

Several experimental studies have provided strong evidence for the influence of carbonated eclogites on modern oceanic- as well as continental intraplate volcanism (e.g., Dasgupta et al. 2004, 2005, 2006 and references therein). In detail, it has been inferred that carbonated eclogite bodies that enter the convecting upper mantle will release carbonate melts that may account for observed seismological anomalies at 280–400 km depth (Dasgupta et al. 2004), similar to what has been observed for the EVF (Ritter and Christensen 2007). A previous model of Kolb et al. (2012) has proposed high Zr/Hf ratios and a positive co-variation of Zr/Hf with La/Yb ratios in volcanic rocks from the nearby Siebengebirge to result from the presence of residual eclogites. This eclogite pattern reflects the diagnostic fractionation of Zr from Hf in residual Ca-rich almandine, a typical mineral phase within mantle eclogites (cf. Klemme et al. 2002). On average, samples from the ONB-suite and EEVF foidites yield both higher Zr/Hf and La/Yb ratios (Fig. 12D). This observation further illustrates a distinct nature of ONB-suite rocks compared to the EEVF and F-suite (also high La/Yb but low Zr/Hf) and tentatively suggests, in combination with the Os isotope data, a more pronounced involvement of recycled eclogitic material and residual garnet for the ONB-suite and some EEVF foidites.

The involvement of distinct, metasomatically overprinted lithospheric mantle sources has been inferred by substantially elevated Nb/Ta ratios (15.9–23.5) in mafic CEVP samples that exceed compositions reported of OIBs (Nb/Ta = ~15–16; Pfänder et al. 2012). Furthermore, Nb/Ta in EVF lavas show positive co-variations with Lu/Hf and Zr/Hf ratios, and negative co-variations with Zr/Nb ratios (Fig. 12A–D). Our data reveal that most samples from the EEVF and the F-suite have Nb/Ta ratios broadly similar to those observed in OIBs, whereas ONB-suite samples and some foidites from the EEVF trend towards substantially elevated Nb/Ta and Zr/Hf ratios. A similar correlation has been found for volcanic rocks from the Vogelsberg, where Pfänder et al. (2012) proposed the involvement of carbonatitic material or remelting of carbonatized peridotite which are both characterized by elevated Nb/Ta and Lu/Hf ratios of ≥20 and ≥0.1, respectively. These features are explained by extreme depletions of Zr and Hf relative to the heavy rare earth elements (HREE) and only moderate depletions of Nb and Ta relative to the light rare earth elements (LREE) in carbonatites (e.g., Pfänder et al. 2012 and references therein). Additionally, it has been previously shown by Nb/Ta and Zr/Hf ratios in kimberlites (e.g., by Aulbach et al. 2011) that carbonatite metasomatism can substantially influence the HFSE inventory of (sub-) lithospheric mantle domains. This is also in accord with previous claims suggesting that European melilitites originate from the interaction of CO_2_–H_2_O-enriched partial melts that form carbonated peridotite layers at the base of the lithosphere (e.g., Wilson et al 1995). Beyond the lithosphere, carbonatite metasomatism has also been shown to occur within the asthenosphere (e.g., Hoernle et al. 2002) which could also explain the HFSE patterns within ONB-suite rocks, that indicate a greater contribution of garnet-bearing, eclogitic mantle sources. Importantly, carbonated melts from carbonated eclogite (Dasgupta et al. 2004) might have metasomatized surrounding mantle material, also explain the observed HFSE systematics for the ONB melts.

Conclusively, our results are in good agreement with a polybaric melting model, where EVF melts may derive from both asthenospheric and lithospheric sources. The proportion of lithospheric components varies, with ONB suite rocks marking greater contributions of the asthenospheric endmember. While higher average depths of partial melting and radiogenic isotope compositions identify a major contribution of a FOZO-type mantle source to ONB-suite volcanism, F-suite and EEVF trace-element and radiogenic isotope compositions are better explained by an enhanced contribution of lithospheric sources and shallower melting depths. Based on high Nb/Ta, Zr/Hf, La/Yb, and radiogenic ^187^Os/^188^Os compositions, our data can now provide evidence for the presence of residual carbonated eclogite components within the upper mantle underneath the CEVP which may be the source for the carbonatite metasomatism of the European lithosphere identified in CEVP melts (Fekiacova et al. 2007; Kolb et al. 2012; Pfänder et al. 2012). While eclogitic components have shown to be stable in rising mantle plumes (e.g., Shi et al. 2022), our results also allow models, where the ONB-suite represents partial melts generated from a metasomatically overprinted TBL at the base of the European lithosphere as previously proposed (Wilson et al. 1995).

## Spatial and temporal constraints on EVF volcanism

The prevailing SiO_2_–undersaturated volcanism within the EVF has emerged during distinct periods that were initiated during the Tertiary (HEVF at ~44–35 Ma; Fekiacova et al. 2007) and had intermittently occurred until the Holocene (WEVF, ~700 Ma to 10.8 ka; EVF, 500 Ma to 12.9 ka; Schmincke 2007). Previous studies have suggested that the two discrete groups of WEVF lavas are coupled to seismological density contrasts of the asthenosphere (Ritter and Christensen 2007; Mertz et al. 2015). In detail, a study by Mertz et al. (2015) has claimed that the areal extent of ONB-suite volcanism (< 80 ka) is in good agreement with a surface projected P-wave anomaly that is thought to reflect a mantle plume underneath the EVF (Ritter and Christensen 2007). In contrast, volcanic edifices that are compositionally referred to as F-suite are located outside the zone of surface projected P-wave anomalies (Mertz et al. 2015). From seismological interpretations, it was proposed by Mertz et al. (2015) that ONB-suite volcanic rocks have incorporated more plume-derived asthenospheric components compared to F-suite rocks. Volcanic rocks from the EEVF broadly overlap with F-suite compositions, suggesting that they also have incorporated less asthenospheric material, irrespective of their degree of silica undersaturation. In general, our newly obtained radiogenic isotope and trace element data confirm these previously reported temporal, spatial, and compositional differences between EEVF, F-, and ONB-suite magmatism. However, Mertz et al. (2015) suggested that volcanism within the WEVF prior to 80 ka must have interacted with lithospheric mantle domains that were no longer available for younger, ONB-suite volcanism. While this may hold true for the WEVF, temporal and compositional overlap of F-suite and EEVF magmatism and the distinct isotopic nature of ONB-suite lavas rather suggest that both mantle domains are still present and contribute to partial melting underneath the EVF. Likewise, the trace-element and Os isotope compositions of ONB samples analyzed within this study require contributions from eclogitic domains.

The data presented here allow some new constraints on the temporal evolution of magmatism within the CEVP. Broadly similar to the model of Mertz et al. (2015), previous studies by Haase et al. (2004) and Jung et al. (2006) have suggested spatial, temporal and compositional heterogeneities to result from discrete magmatic pulses. In detail, the study by Jung et al. (2006) showed that the variability of ^206^Pb/^204^Pb ratios within Tertiary volcanics can be tied to distinct eruptive periods: 39 Ma (Hocheifel) with ^206^Pb/^204^Pb = ~19.0, 37 Ma to 20 Ma (Hocheifel, Siebengebirge and Westerwald) with ^206^Pb/^204^Pb = 19.3–19.7 and 20–10 Ma (Vogelsberg, Hessian Depression) with ^206^Pb/^204^Pb = 18.8–19.3 by combining temporal constraints from Haase et al. (2004) and geochemical compositions of newly measured volcanic rocks from the HEVF. As a potential explanation, Haase et al. (2004) and Jung et al. (2006) suggested the elevated ^206^Pb/^204^Pb to result from enhanced contributions of asthenospheric melts, likely reflecting plume-derived material, whereas lower ^206^Pb/^204^Pb are attributed to result from an EM I-like reservoir, most likely the lithospheric mantle (Jung et al. 2006). Likewise, Binder et al. (2023) report two distinct age groups in the southern CEVP, which they relate to temporal changes in lithospheric composition.

**Fig. 13:**
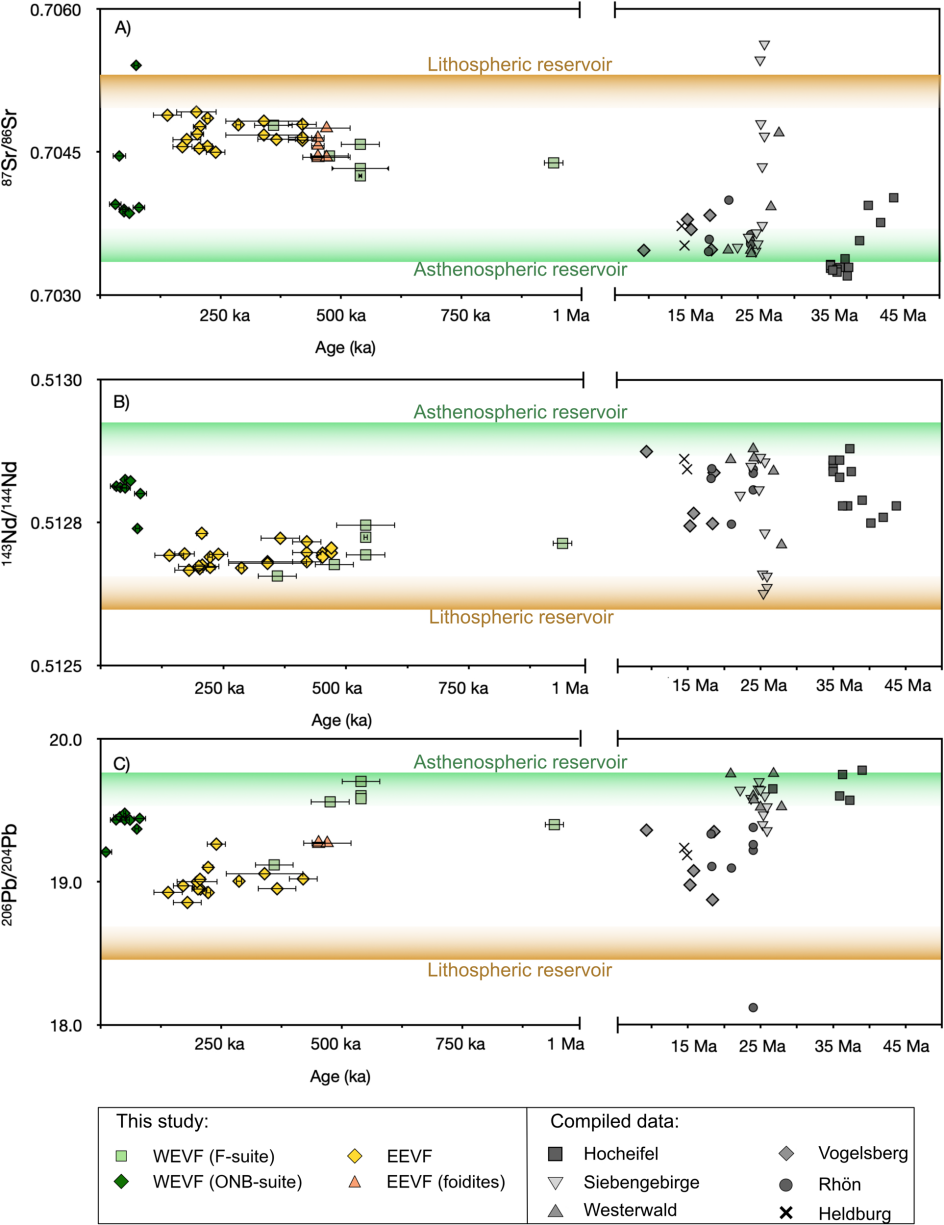
In ^87^Sr/^86^Sr-, ^143^Nd/^144^Nd and ^206^Pb/^204^Pb vs. age diagrams, samples from the CEVP display temporal chemical changes, indicating that available mantle sources changed with time (ages and isotope compositions of Tertiary volcanic rocks: Haase et al. 2004; Jung et al. 2006; Nowell et al. 2006; Fekiacova et al. 2007; Kolb et al. 2012; Mayer et al. 2013; Schneider et al. 2016). For age references on volcanic edifices investigated in this study see Nowell et al. 2006)

While it has already been noted by Jung et al. (2006) that the high ^206^Pb/^204^Pb samples from the youngest Quaternary Eifel volcanism (Wörner et al. 1986) rather resemble an asthenospheric endmember, our data combined with previous age data (Nowell et al. 2006 and references therein; Mertz et al. 2015) now tentatively indicate that the overall compositional–temporal trends may continue throughout the Quaternary (Fig. 13). In detail, distinct pulses of asthenospheric, plume-like material can be identified based on radiogenic Pb and Nd and unradiogenic Sr isotope compositions (Fig. 13) and seem to occur at around ~35 Ma (Hocheifel), ~25 Ma (Siebengebirge, Westerwald), 500 ka (some samples of the F-suite, EEVF) and <80 ka (ONB-suite, EEVF). In contrast, enhanced contributions of lithospheric sources are indicated by radiogenic Sr and unradiogenic Nd and Pb isotope compositions (Fig. 13) and are found for volcanism older than 35 Ma (Hocheifel), at around ~15 Ma (Vogelsberg) and around ~250 ka (EEVF, F-suite). Figure 13 C illustrates that some samples from the F-suite plot at slightly higher ^206^Pb/^204^Pb at about 500 ka, potentially indicating an increasing influence of asthenospheric material. Collectively, by now providing an extensive radiogenic isotope and trace-element dataset for Quaternary volcanic rocks of the CEVP, our study provides further evidence for discrete asthenospheric and lithospheric melt sources underneath the CEVP that now can be traced into the Quaternary.

## Conclusions

Our study provides a new comprehensive trace element and radiogenic isotope dataset for volcanic rocks from the Quaternary Eifel volcanic field (EVF). The data can provide the following new constraints on the origin and source composition of magmatism beneath western Germany and the Cenozoic European Volcanic Province (CEVP) in general:

1. The majority of EVF samples investigated are mafic, primitive volcanic rocks (Mg# ≥ 57) that have undergone only limited fractional crystallization of olivine, clinopyroxene, and amphibole. Plagioclase fractionation is mostly insignificant. Volcanic rocks in the WEVF are more mafic than in the EEVF.
2. Our newly obtained radiogenic isotope data are in good agreement with previous Sr–Nd–Pb isotope studies (Wörner et al. 1986) and mirror the previous petrogenetic subdivision of Mertes and Schmincke (1985) that was based on major elements. While samples from the EEVF and the F-suite of the WEFV display near BSE-like Sr–Nd–Hf isotope compositions, ONB suite lavas show less radiogenic Sr and more radiogenic Hf–Nd compositions similar to the Tertiary HEVF. Further, ONB- and F-suite volcanic rocks and samples from the EEVF can be clearly distinguished based on their distinct Pb isotope compositions. Samples from the EEVF and the F-suite display a linear mixing array, with F-suite samples defining the most radiogenic- and samples from the EEVF defining the unradiogenic end of this Pb isotope array.
3. EC-AFC modelling of Sr–Nd isotope compositions for mafic samples and phonolites from the Rieden Complex can now rule out significant contributions of assimilated lower- or upper-crustal material. Therefore, we conclude that their radiogenic isotope compositions reflect the compositions of their parental magmatic sources.
4. Combined trace-element and radiogenic isotope compositions of ONB-suite samples are in good agreement with results from batch melting models suggesting a hybrid composition formed through mixing 10% of a FOZO-like melt with 90% of a DMM-like melt, similar to melts from the Tertiary HEVF.
5. Partial melting of an enriched, FOZO/EAR-like asthenospheric mantle endmember cannot account for the range of elevated Nb/Ta, Zr/Hf, La/Yb, Ce/Pb, and K*/K ratios observed in EVF lavas. As indicated by radiogenic Sr–Nd–Pb isotope compositions, the admixture of melts from lithospheric mantle sources and carbonated eclogite domains to asthenosphere-derived melts is required for all compositional groups.
6. Elevated Nb/Ta and Lu/Hf at variable ^187^Os/^188^Os can now identify mantle sources that have been overprinted by carbonatite metasomatism (Pfänder et al. 2012). Based on elevated Zr/Hf, La/Yb, Lu/Hf, and Nb/Ta in combination with radiogenic ^187^Os/^188^Os and the decrease of ^187^Os/^188^Os with increasing Re/Os, we therefore suggest the presence of carbonated eclogite sources for the EVF. This carbonated eclogite domain may also be the source for carbonatite metasomatism identified elsewhere within the CEVP (Pfänder et al. 2012; Kolb et al. 2012; Dasgupta et al. 2004).
7. Combined geochemical and temporal constraints suggest that CEVP volcanism in central and western Germany has resulted from compositionally distinct magmatic pulses that now can be extended into the Quaternary. While a lithospheric component must have contributed at variable extent to all eruptive periods, an influence of an asthenospheric, FOZO-like mantle source is predominantly found for the ONB-suite of our sample set and distinct eruptive periods of Siebengebirge, Westerwald, and Hocheifel volcanism.
8. Although we can neither fully exclude nor confirm the presence of a mantle plume beneath the EVF, our results also allow for models, where the ONB-suite represents partial melts generated from a metasomatically overprinted TBL at the base of the European lithosphere as previously proposed (Wilson et al. 1995).

### Supplementary Information

Below is the link to the electronic supplementary material.

Supplementary file1 (PDF 1892 KB)

Supplementary file2 (XLSX 101 KB)

## Data Availability

All data generated or analyzed during this study are included in this published article (and its Supplementary Information files).
