# Supplementary material for: Petrogenesis of volcanic rocks from the Quaternary Eifel volcanic fields, Germany: detailed insights from combined trace-element and Sr–Nd–Hf–Pb–Os isotope data
Source: Contrib Mineral Petrol. 2024 May 9;179(6):57. doi: 10.1007/s00410-024-02137-w (PMC11081932; doi:10.1007/s00410-024-02137-w)
Supplement: Supplementary file 1 — Supplementary file1 (PDF 1892 KB) [file 410_2024_2137_MOESM1_ESM.pdf]

Supplementary file

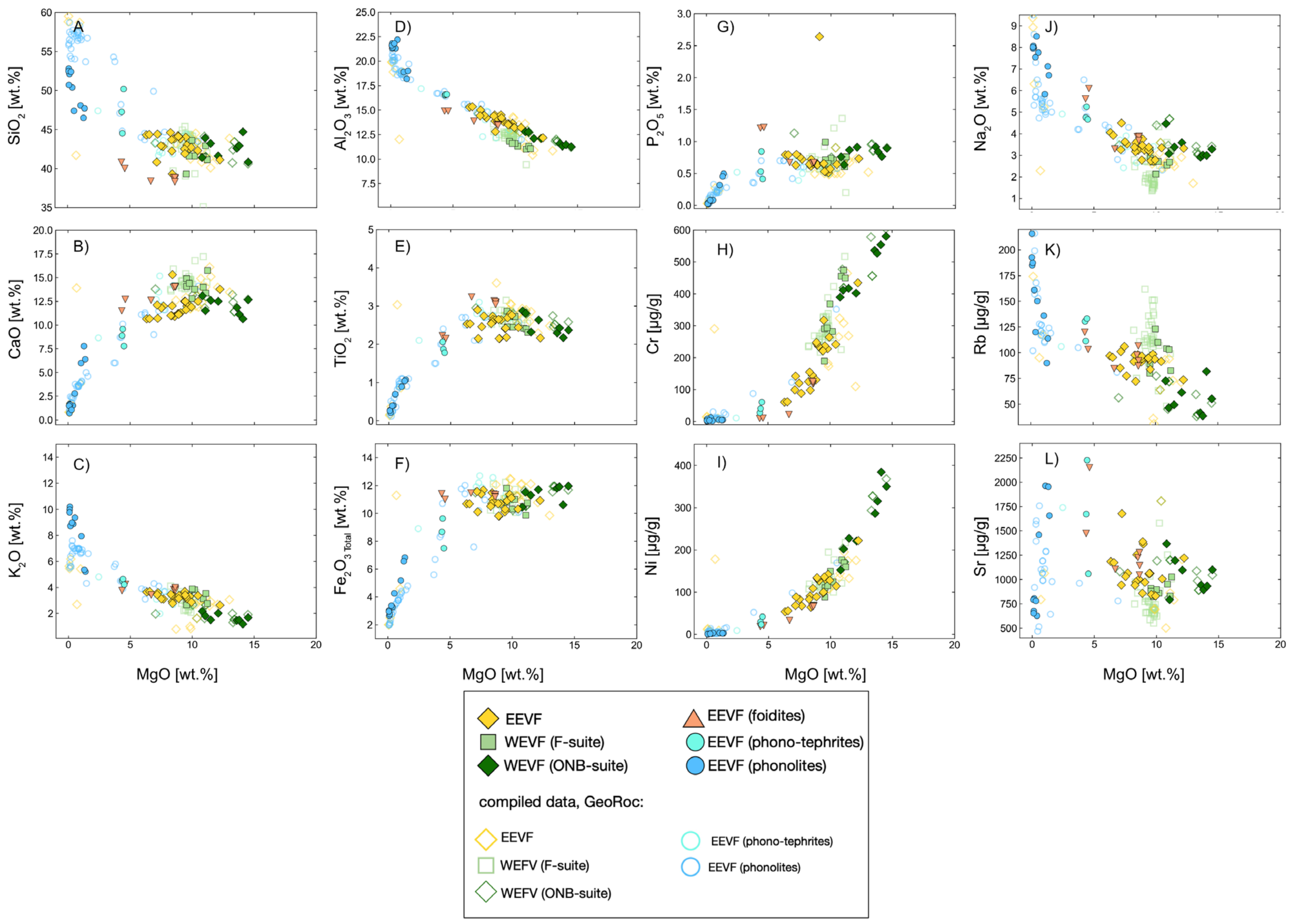

Suppl. figure 1: Plots of selected major element oxides vs. MgO (A-H) variation diagrams illustrating magmatic differentiation trends. Samples of both fields show negative co-variations of SiO_2, Al_2O_3, K_2O, and Na_2O and positive co-variations of CaO, TiO_2 and FeO vs. MgO. In plots of selected trace-element vs. MgO (I-L), Cr (I) and Sr are positively correlated with MgO (L) and Ni (J) and Rb (K) display negative co-variations. Samples of the WEVF are characterized by generally higher MgO (wt. %), Ni and Cr concentrations, illustrating the more mafic and rather primitive character, particularly of ONB-Suite volcanic rocks.

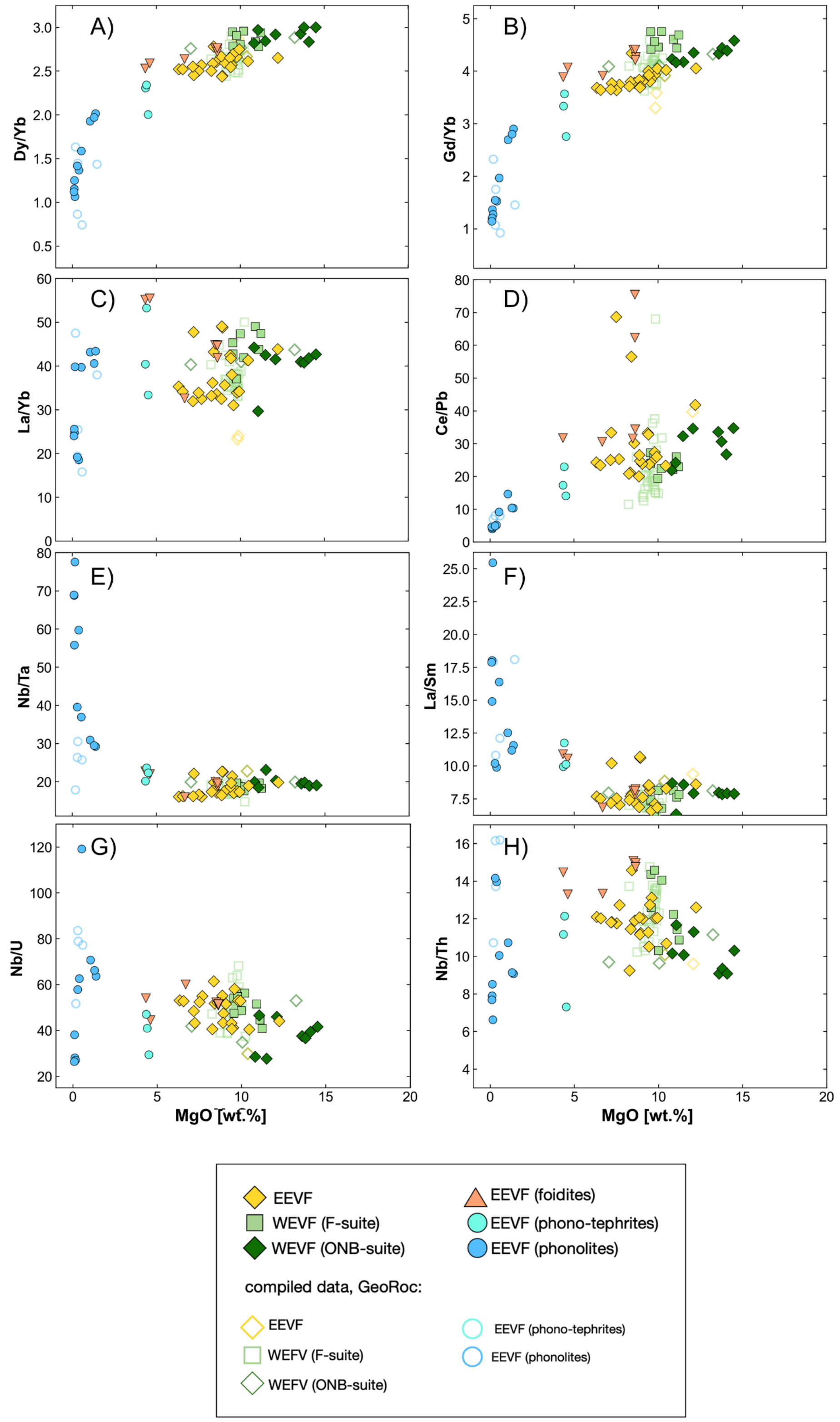

Suppl. figure 2: Selected trace element ratios plotted against MgO [wt. %]. Fractionation of Dy/Yb, Gd/Yb, Ce/Pb and Nb/Ta occurs in differentiated samples, probably due to the fractionation of apatite, sanidine, and titanite. However, less differentiated melts (MgO > 4) were apparently not affected. Additionally, Nb/Th, La/Sm, La/Yb, and Nb/U are only mildly affected by fractional crystallization.

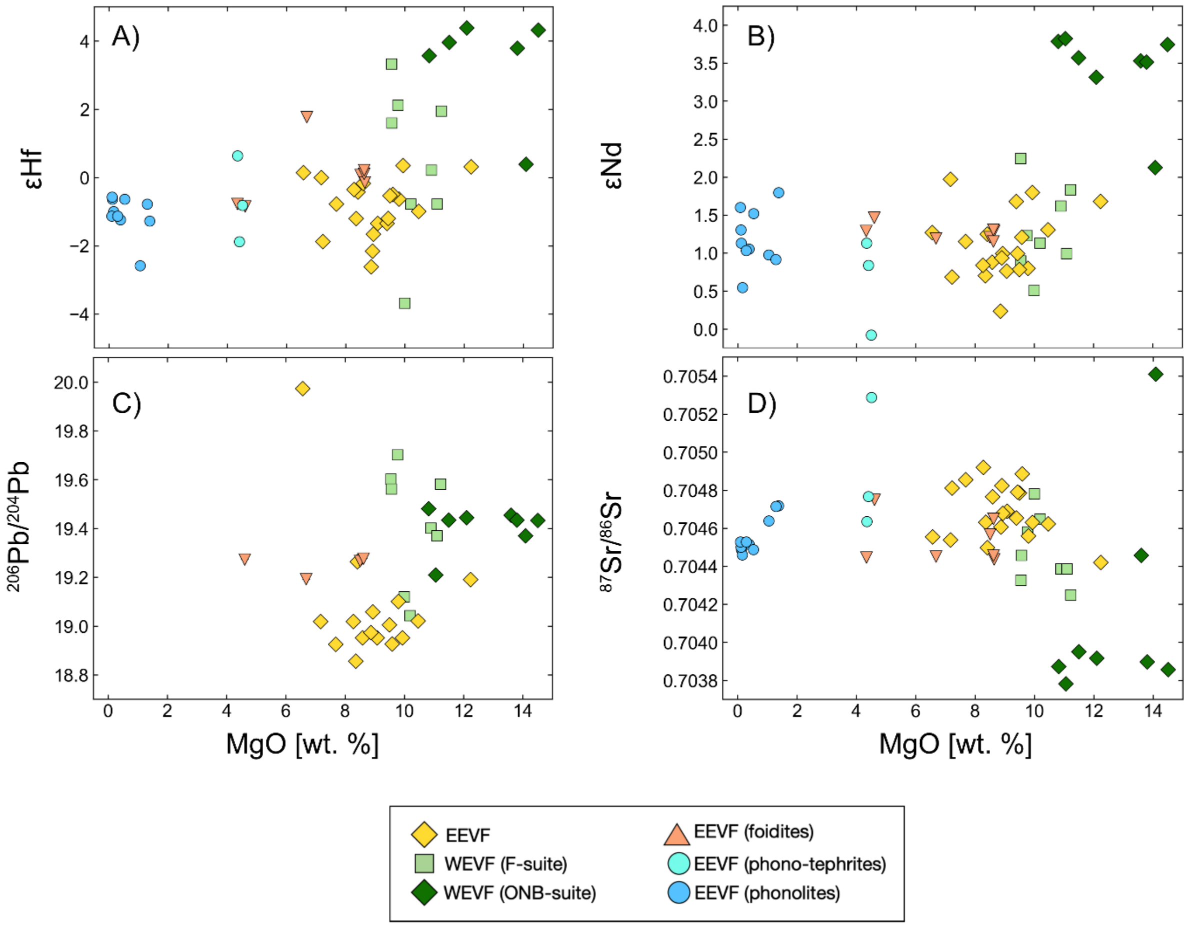

Suppl. figure 3: Radiogenic isotope compositions plotted against MgO. No systematic isotope variation with decreasing MgO concentrations is observed, arguing against significant crustal assimilation.

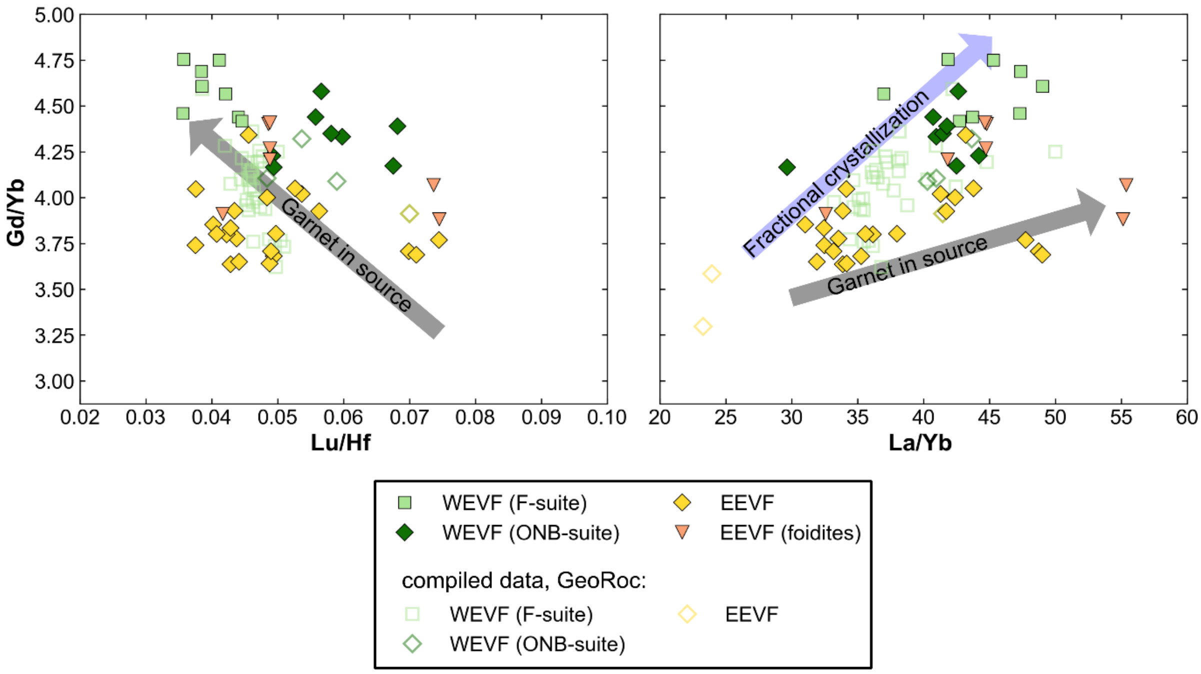

Suppl. figure 4: Plot of Gd/Yb vs Lu/Hf and Gd/Yb vs La/Yb for identification of garnet in the melt source. Differentiated samples (MgO < 4 wt.%) were excluded. It can be observed that samples that display low Lu/Hf also display higher Gd/Yb which is an indication of the presence of garnet in the mantle source.

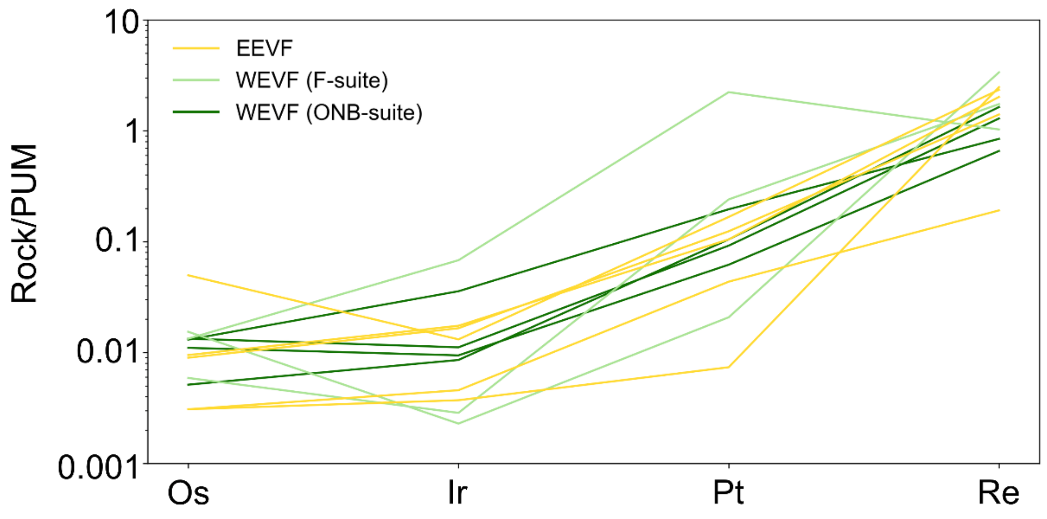

Suppl. figure 5: Primitive upper mantle normalized (Becker et al. 2006) highly siderophile element (HSE) patterns. The samples analyzed within this study display enrichments of the PPGE's compared to the IPGE's, which is typical for primitive upper mantle melts and reflects the incompatible behaviour of Pt and Re during partial melting.

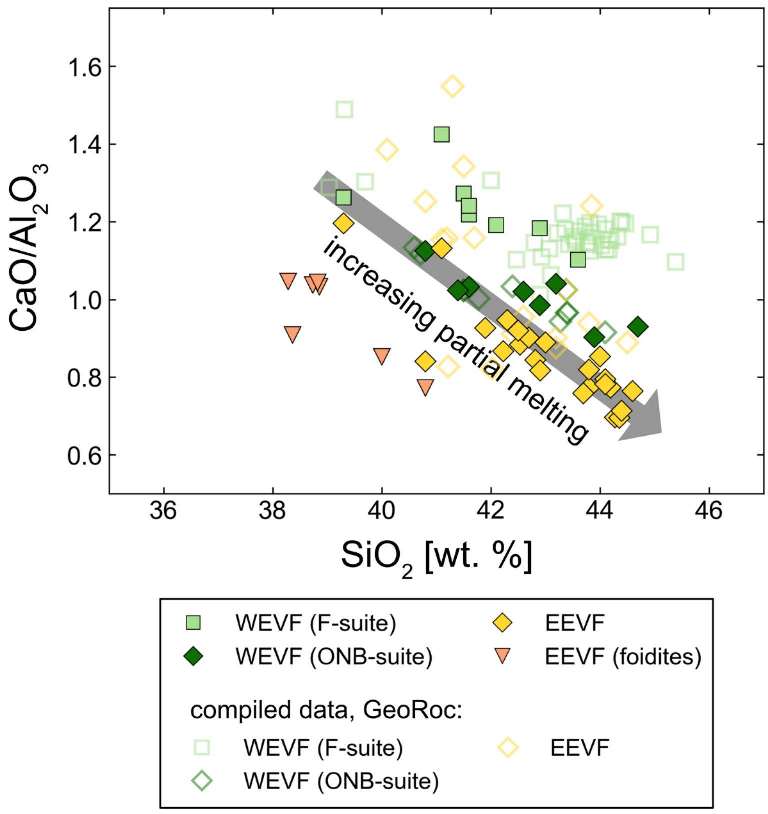

Suppl. figure 6: In CaO/Al_2O_3 vs SiO_2 space, the samples form a linear array, indicating variable depths and degrees of partial melting (Kushiro, 1996). Estimated melting depths and degrees of partial melting are lower for foiditic rocks and increase towards basanites and tephrites.

References

Kushiro, I., 1996. Partial melting of fertile mantle peridotite at high pressures: an experimental study using aggregates of diamond. Geophysical Monograph-American Geophysical Union 95, 109-122.

Becker, H., Horan, M. F., Walker, R. J., Gao, S., Lorand, J.-P., Rudnick, R. L., 2006. Highly siderophile element composition of the Earth's primitive upper mantle: Constraints from new data on peridotite massifs and xenoliths. Geochimica et Cosmochimica Acta 70, 4528-4550.
